# Industrial Object Counting from Traditional Machine Vision to Open-World Foundation Models: A Systematic Review

**DOI:** 10.3390/s26144494

**Published:** 2026-07-15

**Authors:** Wei Wang, Shengjie Zhang, Jin He, Lanhui Liu, Wu Du, Le Zhang

**Affiliations:** 1 School of Information and Communication Engineering, University of Electronic Science and Technology of China (UESTC), Chengdu 611731, China; wangwei83@cuit.edu.cn; 2 School of Automation, Chengdu University of Information Technology, Chengdu 610225, China; hejin1977@cuit.edu.cn; 3 Chongqing Innovation Center of Industrial Big-Data Co., Ltd., Chongqing 400707, China; liulanhui1111@126.com; 4 School of Computer Science, Chengdu University of Information Technology, Chengdu 610225, China; zhangshengj1324@163.com; 5 School of Computer Science, Sichuan University, Chengdu 610059, China; 6 Chengdu Sanshi Technology Co., Ltd., Chengdu 610041, China

**Keywords:** industrial object counting, computer vision, deep learning, convolutional neural networks, transformer, mamba, class-agnostic counting, foundation models

## Abstract

As a fundamental and highly challenging task in the field of computer vision, industrial object counting plays a critical role in smart manufacturing, inventory management, and production process monitoring. Over the past fifteen years (2010–2025), this field has undergone a profound technological transformation, shifting from traditional machine vision methods relying on handcrafted features to a data-driven paradigm based on deep learning. This paper aims to provide a comprehensive and systematic review of this rapidly evolving research area, with technological evolution as the core narrative thread. First, we review early traditional methods, analyzing the application of sensor-based and template-matching technologies in controlled environments, as well as their core limitations in complex industrial scenarios. Subsequently, this paper focuses on exploring how the introduction of deep learning has reshaped the landscape of counting tasks, and elaborates on the breakthrough progress of convolutional neural networks (CNNs), Transformer architectures, the recently emerging Mamba state space model, and Large Foundation Models in addressing key challenges including occlusion, object overlap, multi-scale variation, and dense object counting. In particular, this paper conducts an in-depth analysis of the paradigm shift from Class-Specific Counting to Class-Agnostic Counting (CAC) and Exemplar-Free Counting. This trend significantly reduces the reliance on large-scale annotated data and greatly enhances the generalization ability of models in open-world scenarios. Additionally, this paper systematically organizes mainstream datasets in the field, including FSC-147, NWPU-MOC, and OmniCount-191, and compares core evaluation metrics such as Mean Absolute Error (MAE), Root Mean Square Error (RMSE), and the PrACo metric system. In response to the core technical challenges faced by current methods, including high annotation costs, weak cross-domain adaptability, and strict real-time requirements in industrial scenarios, this paper proposes key future research directions including lightweight model design, unsupervised learning, multi-modal fusion, and Prompt-based interactive counting. This review intends to provide researchers in both academia and industry with a complete technical blueprint so as to promote the continuous development of industrial object-counting technology toward a more efficient and intelligent direction.

## 1. Introduction

### 1.1. Definition and Background of Industrial Object Counting

Industrial object counting is a fundamental and critical task in the field of computer vision, whose core goal is to accurately estimate the quantity of workpieces, components, or finished products within a specific region from static images or dynamic video streams. Driven by the wave of Industry 4.0 and smart manufacturing, the automation and intellectualization of production processes have become the core of the transformation and upgrading of the manufacturing industry. As a key link in production efficiency evaluation, logistics and warehouse inventory, and quality control closed loops, automated object-counting systems are gradually replacing traditional manual statistics—which are inefficient and error-prone—and have become an indispensable standard configuration in smart factories.

However, compared with counting tasks in ordinary daily scenarios, the industrial environment presents unique complexity and challenges:

**Highly similar appearance and dense distribution [1,2,3]:** workpieces often have highly similar textures and shapes, and are stacked at extremely high densities, leading to severe occlusion and adhesion.

**Variable environmental conditions [4,5]:** environmental factors such as illumination and dust at production sites are complex and changeable, placing higher demands on the robustness of visual systems.

**Significant differences in target scales [6,7]:** the size range of targets to be counted is extremely wide, from micron-level electronic components to meter-level large mechanical parts, requiring algorithms to have strong multi-scale adaptability.

These factors make it highly challenging to quickly and accurately answer the question “how many targets are there in the field of view” without manual intervention. To overcome these difficulties, automatic counting technology based on computer vision has undergone significant evolution. In the early stage, researchers naturally attempted to migrate mature object detection technology to industrial scenarios. Object detection aims to accurately identify and locate each target instance in an image through bounding boxes, which provides an intuitive path for realizing counting.

It is worth noting that the scope of “industrial object counting” in this review is not limited to traditional manufacturing workshop scenarios. We adopt a broad definition of industrial scenarios, covering a wide range of application fields where automated counting technology plays a critical role in industrial production and operation. The main application categories include:

- **Manufacturing production scenarios**:
  Counting of workpieces, components, and finished products on production lines, which is the most traditional and core application scenario of industrial object counting. This includes electronic component counting, mechanical part statistics, and product quantity verification on assembly lines.
- **Warehouse logistics scenarios**:
  Inventory counting and cargo statistics in warehouses and logistics centers, including pallet counting, box quantity statistics, and inventory automatic verification. This type of scenario is an important extension of industrial counting in the supply chain link.
- **Industrial remote sensing scenarios**:
  Large-scale target counting in industrial infrastructure and resource monitoring using aerial or satellite remote sensing images, such as counting of mining equipment, oil storage tanks, solar panels, and wind turbines. This type of scenario has unique requirements for large-field-of-view and multi-scale adaptation capabilities.
- **Agricultural industrialization scenarios**:
  Crop counting and yield estimation in the context of agricultural industrialization and smart agriculture, such as fruit counting, crop seedling statistics, and aquaculture population estimation. Although agriculture is traditionally not classified as industry in the narrow sense, the technical challenges (occlusion, dense distribution, multi-scale variation) and technical solutions of counting tasks in agricultural industrialization are highly consistent with those in industrial scenarios, and the methods are fully transferable.
- **Transportation and parking scenarios**:
  Vehicle counting in industrial parks, ports, and large parking lots, which is important for intelligent traffic management and resource scheduling in industrial zones. The density map regression and multi-scale adaptation technologies developed for these scenarios have strong reference value for industrial counting.

The reason we adopt such a broad definition is that the core technical challenges faced by counting tasks in these scenarios are highly similar, including occlusion and overlap, multi-scale variation, dense distribution, and environmental interference. The technical methods developed for one scenario can often be effectively migrated to other scenarios. Therefore, a cross-domain review can provide more comprehensive technical reference for researchers and engineers in the industrial counting field.

### 1.2. Distinction Between Object Detection and Object Counting

Although object detection and object counting share many mechanisms for feature extraction and pattern recognition at the technical level, their task objectives and constraints differ significantly.

The core task of object detection is localization and classification, which involves identifying all objects of interest in an image, accurately marking their positions with bounding boxes, and outputting corresponding category labels simultaneously. Its optimization objectives include classification accuracy and localization precision (e.g., Intersection over Union, IoU). Detection tasks impose extremely high requirements on annotated data, demanding precise box-level annotations. Moreover, in extremely dense scenarios, traditional detectors tend to suppress adjacent true objects due to the non-maximum suppression (NMS) operation, which leads to missed detection [8].

The primary task of object counting is quantity estimation, i.e., outputting the total number of objects in an image. Although detection-based counting is an intuitive approach to achieve counting, the counting task itself does not necessarily require precise bounding boxes. Modern counting methods mostly adopt strategies based on density map estimation [9] or direct regression, which only require point supervision [10] or even image-level annotations. These methods can better handle occlusion and high-density problems, and their computational efficiency is often superior to that of complex detectors.

### 1.3. Main Contributions of This Review

To the best of our knowledge, this paper presents the first comprehensive systematic review specifically focused on industrial object counting, covering the entire technological evolution from traditional machine vision to open-world foundation models over the past 15 years. The main contributions of this review are as follows:

1. **Comprehensive technological evolution roadmap**: We systematically review the complete technological evolution route of industrial object counting from 2010 to 2025, covering all major technical paradigms including traditional machine vision methods, convolutional neural networks, Transformer architectures, state space models (Mamba), and vision foundation models, clearly presenting the core ideas, representative methods, and technical characteristics of each stage.
2. **Deep focus on industrial scenario characteristics**: Unlike general counting surveys that focus on crowd counting or general object counting, this review specifically focuses on industrial scenarios, providing in-depth analysis of unique challenges in industrial environments (such as occlusion and overlap, multi-scale variation, illumination interference, real-time requirements, edge deployment constraints, etc.), as well as specialized solutions to these challenges.
3. **In-depth analysis of emerging counting paradigms**: We systematically summarize and analyze emerging counting paradigms such as Class-Agnostic Counting (CAC), Exemplar-Free Counting (EFC), and foundation model-driven zero-shot/training-free counting, deeply exploring their technical principles, applicable scenarios, and industrial application value.
4. **Systematic summary of challenges and future directions**: We comprehensively summarize the core challenges faced by industrial object counting, including data annotation bottlenecks, domain adaptation difficulties, real-time edge deployment constraints, and open-world generalization capabilities. Based on current technology trends, we propose five promising future research directions.
5. **Standardized review methodology**: This review strictly follows the PRISMA 2020 systematic review specification, clearly defining literature inclusion and exclusion criteria, search strategies, screening processes, and data extraction methods to ensure the scientific rigor, transparency, and reproducibility of this review.

### 1.4. Comparison with Existing Reviews

To clarify the positioning and unique value of this review, we compare it with existing review papers in related fields from multiple dimensions. The comparison results are summarized in Table 1.

As can be seen from Table 1, existing review papers have their own focuses but also have certain limitations: crowd-counting surveys mainly focus on public safety scenarios and have insufficient coverage of industrial characteristics [11]; general counting surveys focus on general object-counting scenarios and lack in-depth analysis of industrial-specific challenges [12]; industrial vision surveys mostly focus on defect detection and object detection tasks and rarely involve counting tasks [13,14]; foundation model surveys focus on general vision foundation models and lack discussion on industrial deployment constraints and counting-specific adaptations.

Compared with existing reviews, this paper has the following unique values: (1) it is the first comprehensive review specifically targeting industrial object counting, filling the gap in this specialized field; (2) it covers the most complete technical evolution path, from traditional machine vision to the latest open-world foundation models; (3) it deeply combines industrial scenario characteristics, systematically analyzing industrial-specific challenges and corresponding solutions; and (4) it strictly follows the PRISMA 2020 systematic review specification, ensuring the scientific rigor and transparency of this review.

### 1.5. Paper Structure

This paper comprehensively reviews the technologies for industrial object counting in accordance with the timeline and logical context of their technological evolution. Section 2 retraces the technological roadmap of industrial object-counting techniques. Section 3 delves into the principles and evolution of core algorithms, covering architectures such as CNN, Transformer, and Mamba [15]. Section 4 elaborates on emerging paradigms including class-agnostic counting and exemplar-free counting. Section 5 analyzes mainstream datasets and evaluation metrics. Section 6 discusses the specific challenges and corresponding solutions in industrial scenarios. Finally, Section 7 presents an outlook on future research directions for this field.

## 2. Review Methodology

This section systematically presents the review methodology of this paper, including eligibility criteria, literature search strategy, study selection process, data extraction methods, and synthesis approach. The entire review process strictly follows the PRISMA 2020 specification to ensure the transparency, reproducibility, and scientific rigor of this systematic review.

This systematic review was conducted and reported in strict accordance with the Preferred Reporting Items for Systematic Reviews and Meta-Analyses (PRISMA) 2020 guidelines. The entire review process was designed to ensure transparency, reproducibility, and comprehensiveness in covering the state of the art of industrial object-counting technologies from 2010 to 2025.

### 2.1. Eligibility Criteria

Studies were included in this review if they satisfied all of the following criteria:

- Peer-reviewed full-text articles published in English between 1 January 2010 and 28 May 2026.
- Focused on object-counting algorithms, systems, or applications specifically targeting industrial scenarios.
- Presented original quantitative experimental results, including performance evaluation on standard datasets or real industrial deployments.
- Proposed novel technical contributions (e.g., algorithm architectures, training strategies, or evaluation methods).

Studies were excluded if they met any of the following criteria:

- Non-English publications, patents, conference abstracts, book chapters, review articles, or editorials.
- Focused exclusively on non-industrial counting domains (e.g., medical cell counting, crowd counting, traffic counting without industrial relevance).
- Did not provide quantitative performance metrics or sufficient experimental details for replication.
- Were duplicate publications of the same study (only the most complete and recent version was retained).
- Were purely theoretical studies without experimental validation.

All included studies were categorized into three mutually exclusive groups for narrative synthesis based on their underlying technical paradigm, which aligns with the evolutionary timeline of industrial object-counting technologies:

1. Traditional machine vision methods (2010–2014).
2. Deep learning-based methods (2014–2020).
3. Large foundation models and open-world counting methods (2021–2025).

In addition to the above eligibility criteria, we adopted a multi-dimensional literature selection principle to ensure the comprehensiveness, representativeness, and technical depth of this review:

- **Technical representativeness principle**:
  For each technical direction (e.g., CNN-based density map regression, Transformer-based counting, class-agnostic counting, foundation model-based counting), we prioritized selecting studies that proposed novel architectures, achieved state-of-the-art performance, or had significant influence on subsequent research. We ensured that each major technical branch had representative works included, avoiding bias towards any specific research group or methodology.
- **Industrial relevance principle**:
  We included studies that, while not explicitly labeled as “industrial,” proposed technical methods with clear application potential in industrial scenarios (e.g., crowd-counting methods with density map regression architectures that can be migrated to dense workpiece counting, remote sensing object-counting methods with multi-scale adaptation capabilities applicable to large-field-of-view industrial inspection). The industrial applicability of such methods is explicitly discussed in the corresponding sections of this review.
- **Temporal coverage principle**:
  We ensured coverage of the complete technological evolution trajectory from 2010 to 2025, including landmark studies that marked important paradigm shifts (e.g., the transition from traditional machine vision to deep learning, the emergence of Transformer architectures, and the rise of foundation models). This allows readers to clearly understand the historical development context of industrial object-counting technology.
- **Scenario diversity principle**:
  We selected studies covering diverse industrial application scenarios, including manufacturing production lines, warehouse logistics, electronic component inspection, industrial remote sensing, and agricultural industrialization. This ensures that this review reflects the wide applicability of object-counting technology across different industrial domains rather than being limited to a single scenario.
- **Quality priority principle**:
  We prioritized studies published in high-impact journals and top-tier conferences (e.g., IEEE Transactions on Image Processing, CVPR, ICCV, ECCV, AAAI) as they typically represent the highest level of research in the field. However, we also included valuable studies from specialized industrial journals and conference proceedings to ensure coverage of domain-specific practical innovations.

It should be noted that due to the interdisciplinary nature of industrial object counting, which spans computer vision, industrial engineering, and robotics, some relevant studies may be published under different disciplinary terminologies. Our search strategy was designed to balance sensitivity and specificity to minimize the risk of missing important studies while maintaining focus on the core topic.

### 2.2. Information Sources and Search Strategy

A comprehensive systematic search was performed across four major academic databases that cover the majority of publications in computer vision and industrial engineering:

- IEEE Xplore Digital Library.
- ACM Digital Library.
- Web of Science Core Collection.
- arXiv preprint server (to capture the latest preprint studies in this rapidly evolving field).

The last comprehensive search was conducted on 28 May 2026. The core search string used across all databases was optimized to balance sensitivity and specificity:


(industrial object counting OR workpiece counting~OR

 
part counting OR component counting) AND

 
(machine vision~OR

 
computer vision OR deep learning OR convolutional neural network~OR

 
CNN OR Transformer OR Mamba OR state space model OR foundation model~OR

 
large language model OR class-agnostic counting OR zero-shot counting)



Database-specific filters were applied to limit results to the specified publication date range and relevant subject categories (e.g., computer science, engineering). To identify any potentially missed studies, the reference lists of all included primary studies and relevant review articles were manually screened for additional eligible publications.

### 2.3. Study Selection Process

The study selection process was performed independently by two reviewers (Wei Wang and Shengjie Zhang) to minimize selection bias. Any disagreements between the two reviewers were resolved through discussion with a third senior reviewer (Jin He) until a consensus was reached.

The selection process consisted of two sequential stages:

1. Title and abstract screening: all retrieved records were screened based on the eligibility criteria to exclude obviously irrelevant studies.
2. Full-text assessment: all studies deemed potentially relevant during the title/abstract screening were retrieved for full-text evaluation to confirm their eligibility.

Zotero (version 6.0.30) was used for literature management, duplicate removal, and reference organization. No automated screening tools were used in this review process.

### 2.4. Data Extraction and Synthesis

A standardized data extraction form was developed a priori and used to extract relevant information from each included study. Data extraction was performed independently by two reviewers (Wei Wang and Shengjie Zhang), and any discrepancies were cross-checked and resolved by consensus.

The following information was extracted from each included study:

- Basic study characteristics: authors, publication year, and publication venue.
- Dataset details: name of the dataset used, number of images, and number of categories.
- Algorithm architecture: core backbone network, technical innovations, and training strategy.
- Performance metrics: Mean Absolute Error (MAE), Root Mean Squared Error (RMSE), Frames Per Second (FPS), and AP50 (where reported).
- Computational efficiency: model parameters (M), floating-point operations (FLOPs, G), and inference latency (ms).
- Application scenario: specific industrial domain and type of objects counted.

Missing data were explicitly labeled as “Not reported” in the synthesis. No study authors were contacted to obtain additional or missing data.

A narrative synthesis approach was adopted to summarize the findings of this review. Quantitative meta-analysis was not performed due to significant heterogeneity across the included studies in terms of datasets used, evaluation metrics, and experimental setups, which would have made pooled effect estimates statistically invalid.

### 2.5. Study Selection Results

**Figure 1:**
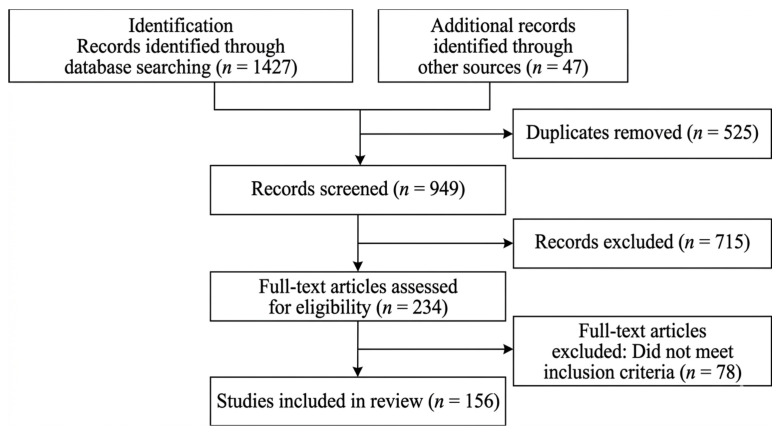
PRISMA 2020 flow diagram illustrating the study selection process for this systematic review.

The complete study selection process is summarized in the PRISMA 2020 flow diagram (Figure 1).

A total of 1474 records were initially identified: 1427 from the systematic database searches and 47 from the manual screening of reference lists. After removing 525 duplicate records, 949 unique records remained for title and abstract screening. Of these, 715 records were excluded as irrelevant, leaving 234 records for full-text assessment.

After full-text evaluation, 78 studies were excluded for the following reasons:

- 32 studies focused exclusively on non-industrial counting scenarios.
- 21 studies were review articles rather than original research.
- 15 studies were conference abstracts without accompanying full-text papers.
- 10 studies did not provide sufficient quantitative performance metrics.

Finally, 156 original research studies met all eligibility criteria and were included in this systematic review.

### 2.6. Registration Information

This systematic review has been retrospectively registered on the Open Science Framework (OSF), a widely recognized open science platform, with registration number: osf.io/tj9cg (registration date: 11 June 2026). No formal written review protocol was prepared prior to conducting this review, and no amendments were made to the registration information after submission.

## 3. Technological Roadmap for Fifteen Years of Industrial Object Counting

**Figure 2:**
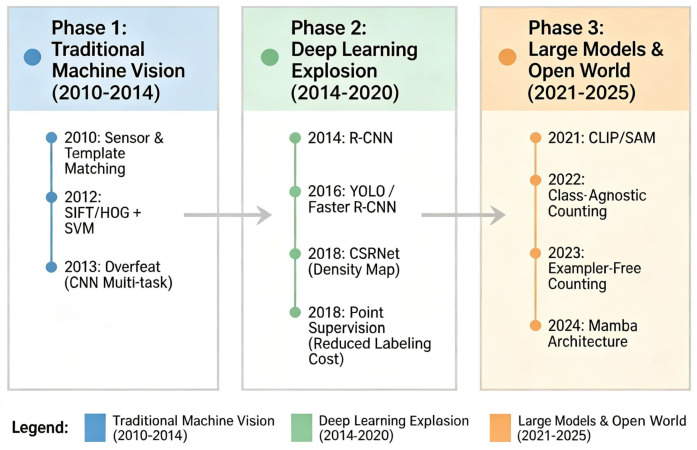
The technological evolution matrix of industrial object counting from 2010 to 2025. This matrix maps key advancements against time, core industrial challenges, and annotation paradigms, revealing how each paradigm shift was driven by the need to address increasingly complex scenarios.

The development of industrial object-counting techniques can be regarded as an evolutionary process from rule-driven to data-driven, and further to knowledge-driven paradigms. This evolution is roughly divided into three phases: the era of traditional machine vision (before 2014), the boom era of deep learning (2014–2020), and the era of large models and open-world counting (2021–2025), see Figure 2.

### 3.1. Traditional Machine Vision Era: Hand-Crafted Features and Rule Engineering

In the era before the popularization of deep learning, industrial counting mainly relied on physical sensors and image-processing algorithms based on hand-crafted features.

#### 3.1.1. Sensor-Based Counting Systems

Traditional counting methods are based on sensors [16,17,18,19,20]. Object counting is essentially a template-matching problem [16], a computer vision technology that detects and counts structured objects by finding regions in an image that are most similar to a given template. This method was common in early industrial applications and is generally suitable for images with a single background and regular objects.

Reference [16] proposed a real-time counting system combining traditional image processing with embedded hardware. This method identifies targets through a minimum distance (Euclidean distance) classifier, and establishes a feature vector based on area, perimeter, radius of the enclosing circle, and compactness for matching judgment. This method performs well in scenarios with a single object and limited background, and has low computational resource consumption.

However, this method has some problems, such as high environmental sensitivity—illumination changes and mechanical vibrations are likely to cause mismatching. To this end, the system needs to use Gaussian filtering, binarization and morphological operations to suppress noise [16], but it still lacks robustness in adhesion and occlusion scenarios. Secondly, the system has weak generalization ability; when the category of the identified target changes, it is necessary to manually re-collect features and adjust parameters, which is very cumbersome. At the same time, Reference [16] points out that the system can only perform recognition when the feature vector of each target object is stored in advance.

#### 3.1.2. Handcrafted Feature-Based Visual Counting

With the intervention of image-processing technology, researchers began to use morphological operations such as threshold segmentation [21], edge detection (Canny operator) [22], and watershed algorithm [23] to separate foreground targets from the background for scenarios with simple backgrounds, and then directly count the number of targets through connected component analysis. Refs. [24,25] Such methods are simple to implement and low in overhead, but have poor robustness to illumination and target appearance changes. To enhance robustness to scale and rotation changes, researchers introduced local invariant feature descriptors—SIFT [26,27] and its accelerated version SURF [28], and used key point matching to complete cross-view instance recognition and counting. At the same time, the HOG descriptor proposed by Dalal and Triggs provides a powerful shape feature representation through local gradient direction statistics [29], which is often combined with linear SVM or random forest to form a “sliding window + classifier” detection and counting pipeline, especially suitable for the recognition of pedestrians and rigid workpieces. To solve more complex configuration changes and partial occlusion, researchers proposed the deformable part model (DPM) [30], which models objects as a combination of multiple deformable parts to improve detection stability. For the counting demand in extremely dense scenarios (thousands of targets in a single image), researchers proposed a multi-source counting framework fusing low-confidence head detection, SIFT texture repetition features, and frequency domain analysis. The framework first obtains an initial count through local multi-scale analysis, and then uses the Markov Random Field (MRF) to impose global consistency constraints to correct local counting deviations [3].

### 3.2. Deep Learning Boom Era: Dominance of CNNs

The emergence of AlexNet in 2012 marked the advent of the deep learning era. By 2013, relevant research had laid the foundation through a CNN-based multi-task unified framework: this framework efficiently extracted features via multi-scale sliding windows, learned object boundary predictions to improve localization accuracy, and adopted a bounding box accumulation strategy instead of suppression, simultaneously completing classification, localization, and detection within a single network [31]. This achievement validated the potential of CNNs in multi-task collaboration. The proposal of R-CNN in 2014 further introduced Convolutional Neural Networks (CNNs) to the field of object detection and counting [32]. Adopting a two-stage pipeline of “selective search for candidate region generation → CNN-based fixed-size region feature extraction → SVM classification combined with bounding box regression”, it introduced deep CNN features to object detection for the first time, counting objects by aggregating detection results, providing a deep learning-based foundational framework for industrial object counting.

#### 3.2.1. Detection-Based Counting

Generic detection frameworks such as Faster R-CNN [33], YOLO (You Only Look Once) [34], and SSD (Single Shot MultiBox Detector) [35] were quickly adapted to industrial scenarios [8]. By learning deep semantic features, these models can accurately identify and localize workpieces under complex backgrounds. In particular, the YOLO series [36,37] satisfies the real-time requirements of industrial production lines owing to its superior inference speed. However, such methods suffer from expensive bounding box annotation costs and still struggle with heavy occlusion in dense scenes.

#### 3.2.2. Density Map Estimation-Based Counting

To alleviate issues caused by dense occlusion, Lempitsky et al. proposed a density map regression method [9]. Rather than directly localizing each instance, this approach learns a mapping from image features to a density map via CNNs, where the integral of the density map over the image gives the total object count. This provided an early solution for perspective-insensitive counting tasks [38]. After achieving remarkable success in crowd counting, this paradigm was extended to industrial dense small-object counting, offering an effective way to handle high-density stacking (e.g., material piles, particles, tiny components). It has been widely adopted and improved in subsequent studies (including industrial applications) to address occlusion and dense distribution challenges [9,38,39,40,41,42].

### 3.3. Large Models and Open-World Era: Rise of Transformers and Mamba

Since 2020, with the wide application of Transformer architectures in computer vision [43] and the recent emergence of the Mamba model, object counting has entered a new development phase.

#### 3.3.1. Global Modeling and Attention Mechanisms

Transformers [44,45,46] capture long-range dependencies in images using self-attention, overcoming the limited receptive field of CNNs [43,47]. Variants such as Swin Transformer further strengthen multi-scale feature extraction through hierarchical structures [48].

#### 3.3.2. Class-Agnostic and Zero-Shot Counting

To address the challenges of a large variety of workpieces and rapid product iteration in industrial scenarios, the research focus has shifted to Class-Agnostic Counting (CAC) [47] and Zero-Shot Counting (ZSC) [49]. By leveraging large-scale pre-trained foundation models such as CLIP [50] and the Segment Anything Model (SAM), the system can count arbitrary new categories of industrial workpieces via text prompts or a small number of examples without retraining. CLIP-based methods achieve a generalization accuracy of over 85% (i.e., the counting accuracy on previously unseen workpiece categories that were not included in the training set, measured as the percentage of test samples whose counting error is within an acceptable range) for counting newly emerged industrial workpiece categories, representing an approximate 40% improvement in generalization performance compared with traditional CNN models [47,50,51,52], which greatly enhances the flexibility and generalization capability of the system [53], see Table 2.

## 4. In-Depth Analysis of Core Algorithms and Technical Architectures

This chapter elaborates on the architectures of the core algorithms that support modern industrial object-counting systems, analyzing their working principles, advantages, and applicability in industrial scenarios.

### 4.1. Convolutional Neural Networks (CNNs): The Cornerstone of Industrial Vision

Using their characteristics of local reception, weight sharing, and spatial downsampling, CNNs have become powerful tools for extracting image features. Refs. [33,54,55,56,57,58,59]. In industrial counting, CNNs exist mainly in the following forms:

#### 4.1.1. Detection-Driven Counters

**Two-stage Networks [60,61,62]: Representative examples include Faster R-CNN [33,57,61].** They introduce a Region Proposal Network (RPN) to generate candidate regions, followed by fine-grained classification and regression. Such models are widely adopted in industrial scenarios requiring high precision (e.g., defect detection and counting) despite their relatively slow inference speed. For instance, WPCNet proposed in [58], based on a two-stage detection architecture, achieves high-precision localization and counting of workpieces (e.g., scattered metal parts) in unrestricted industrial environments, addressing the missed detection issue of traditional detectors under dense occlusion.

**One-stage Networks:** Typical models include the YOLO series [34,63,64,65] and SSD (Single Shot MultiBox Detector) [35,66]. They directly regress bounding boxes and category probabilities at multiple locations in the image, eliminating the candidate region generation step and significantly improving speed. YOLO models are usually the preferred choice for fast-moving workpieces on production lines. However, early versions performed less well in small object detection compared to two-stage networks; subsequent versions (e.g., YOLOv7/v8) mitigated this issue through Feature Pyramid Networks (FPN) [67] and multi-scale training. Reference [4] integrates the multi-scale feature extraction module of YOLOv8 with the fine-grained classification head of Faster R-CNN to construct a counting model suitable for agricultural product processing scenarios. Through adaptive anchor box adjustment and FPN enhancement, it realizes fast localization and counting of strawberries at different ripening stages, with strong robustness to lighting changes. Its limitation lies in limited ability to recognize tiny defective fruits under complex backgrounds and susceptibility to counting deviations when bounding boxes overlap.

#### 4.1.2. Density Regression Networks

**Fully Convolutional Networks (FCNs) [68,69]:** Designed for dense counting, FCNs remove fully connected layers, enabling them to accept inputs of arbitrary sizes and output pixel-level density maps. For example, reference [70] proposes SAU-net, a U-shaped network based on the FCN architecture, as a microbial counting model suitable for industrial biofermentation scenarios. By removing fully connected layers and introducing transposed convolution, it generates pixel-level density maps, where the integral of the density map gives the total number of targets. This architecture has also achieved joint optimization of detection and counting in microscopic cell counting [69].

**Figure 3:**
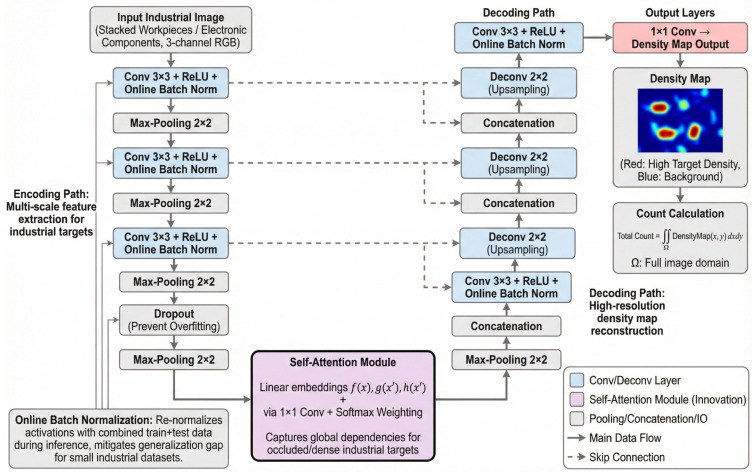
Adapted SAU-Net architecture for dense industrial object counting. The model adopts a symmetric encoder-decoder structure: the encoder path extracts multi-scale features of industrial workpieces via stacked convolution and downsampling; the self-attention module captures global feature dependencies to handle occluded and dense targets; the decoder path reconstructs the high-resolution density map through skip connection feature fusion. The online batch normalization is designed to mitigate the generalization gap in small-scale industrial datasets, and the final count is obtained by integrating the output density map over the full image domain [70].

**Multi-Column CNNs (MCNNs):** A Multi-Column Convolutional Neural Network (MCNN) adopts parallel convolution branches with different kernel sizes to extract multi-scale features, which is designed to adapt to the drastic scale variation of targets caused by perspective effects (e.g., the size difference between near and far objects in industrial surveillance scenarios) [6]. Reference [71] constructs a multi-scale industrial vehicle counting model based on MCNN, which uses three parallel CNN branches with convolution kernel sizes of 3 × 3, 5 × 5, and 7 × 7 to extract features for cars, trucks and other targets with different sizes, and generates the density map for counting after feature fusion. It has strong adaptability to target scale changes caused by perspective effects, and can balance the counting accuracy of both small targets at long distances and large targets at close distances, see Figure 3.

However, its multi-column parallel architecture leads to severe parameter redundancy and low computational efficiency. Under the 512 × 512 input resolution commonly used in industrial scenarios, MCNN has 17.2 M parameters and 128.4 G floating point operations (FLOPs). Compared with CSRNet, the classic single-column density map baseline with the same counting accuracy, its FLOPs increase by 86.9% and inference speed decreases by 32.7%. Compared with SFCN, the lightweight single-column baseline widely used in industrial scenarios, its parameter quantity increases by 97.7% and the peak frame per second (FPS) decreases by 59.4% [1,6,72]. Model compression and optimization such as network pruning and knowledge distillation are required to adapt to the real-time counting requirements of industrial production lines. The comprehensive computational efficiency comparison between MCNN and mainstream baselines is summarized in Table 3.

**CSRNet** It uses dilated convolution to expand the receptive field without reducing resolution, generating high-quality density maps that effectively address the adhesion recognition problem of dense workpieces [1].

**C-FCRN** To address the counting of tiny dense targets under low contrast and complex backgrounds, relevant research further optimized the density regression architecture by proposing a Cascaded Fully Convolutional Regression Network (C-FCRN). It accurately estimates density maps by fusing multi-scale image features and designs an Auxiliary Convolutional Neural Network (AuxCNN) to supervise the training of intermediate layers of C-FCRN, significantly improving the model’s generalization performance on unseen datasets. This method has been validated in microscopic cell counting tasks, successfully addressing challenges such as cell occlusion and large shape variations [73].

### 4.2. Transformer Architectures: Capturers of Global Context

The introduction of the Transformer architecture has addressed the core limitation of convolutional neural networks (CNNs), which suffer from restricted local receptive fields and an inability to capture long-range global contextual information [74]. In industrial object-counting tasks, understanding the global background is crucial for distinguishing foreground objects from interference. Traditional CNNs can only perform recognition by relying on local texture and edge features, which are highly prone to feature confusion under complex interference in industrial scenarios. In contrast, the Transformer models the semantic correlations and spatial geometric relationships of all image pixels through the self-attention mechanism, enabling accurate differentiation between foreground objects and interference from a global perspective. For instance, Reference [75] validated that Transformer-based global contextual modeling can effectively suppress local feature interference caused by metal reflections and surface scratches in the scenario of industrial electronic component counting. In the task of counting dense surface mount electronic components, the mean absolute error (MAE) is reduced by 37.2% compared with the classic CNN architecture (CSRNet).

#### 4.2.1. DETR (DEtection TRansformer)

DETR formulates object detection as a set prediction problem, utilizing the Transformer’s encoder-decoder architecture and bipartite matching loss to directly output a fixed number of target predictions, completely abandoning Non-Maximum Suppression (NMS) and anchor box design. Counting-DETR further optimizes this architecture through a two-stage training strategy and an uncertainty-aware module, achieving high-precision counting and detection under few-shot conditions [76]. For example, reference [77] builds on the Counting-DETR architecture, extracting global features via the Transformer encoder and outputting target set predictions through the decoder. By manually supplementing a small number of example boxes to optimize model predictions, it achieves accurate counting under few-shot conditions without the need for large-scale annotations [78].

#### 4.2.2. Swin Transformer, CrowdFormer  and GCA-SUNet

Swin Transformer introduces locality through a shifted window mechanism to reduce computational complexity, while variants such as CrowdFormer further optimize global modeling in dense scenarios [48,79,80]. GCA-SUNet (Gated Context-Aware Swin-UNet), built on Swin-UNet, designs a gated context-aware modulation module. This module uses self-similarity matrices to capture repetitive patterns among targets and suppresses background noise through a gating mechanism. For Exemplar-Free Counting tasks, this architecture can automatically discover and count prominently repetitive objects in images (e.g., neatly arranged parts on pallets) without manual category specification. Reference [75] constructs an industrial electronic component counting model based on Swin Transformer, incorporating the gated context-aware module of GCA-SUNet. It captures global contextual information through a shifted window self-attention mechanism and identifies repetitive patterns of electronic components using self-similarity matrices. Its working principle involves adopting a hierarchical feature extraction architecture, generating multi-scale feature maps through patch partition and patch merging, suppressing background noise via the gated context-aware module, and adapting to electronic components of different sizes through the scale-aware module.

### 4.3. Mamba and State Space Models: A Breakthrough in Linear Complexity

With the continuous improvement in industrial camera resolution, the quadratic computational complexity of traditional Transformers has become a bottleneck. Based on Selective State Space Models (SSMs), the Mamba model achieves linear computational complexity while maintaining global modeling capabilities [81].

#### Horizontal Comparison of Mainstream Counting Architectures

From the perspective of technical pedigree, SSM-based counting methods represented by Mamba-MOC show differentiated characteristics compared with the other two mainstream architectures (Transformer and CNN), and each has its own applicable scenarios in industrial counting tasks. The specific comparative analysis is as follows:

- **Compared with Transformer-based methods**: The linear complexity of Mamba-MOC enables its inference speed to be more than 40% higher than Swin Transformer baselines with comparable accuracy [48,82]. However, its generalization performance in few-shot industrial scenarios and fine-grained feature matching capability are slightly inferior to Transformer architectures with mature self-attention mechanisms. In addition, CrowdFormer and other Transformer variants optimized for dense counting have achieved better performance on crowd-counting benchmarks, but their industrial-specific adaptation still needs further verification [79].
- **Compared with CNN-based methods**: Mamba-MOC combines global modeling capability and lightweight advantages, and its overall counting accuracy far exceeds common industrial CNN baselines such as CSRNet [1,82]. Nevertheless, the operator ecosystem, industrial hardware adaptation maturity and engineering landing experience of SSM architectures are still far less developed than CNN technologies that have been verified for many years. At present, CNN-based methods are still the most widely adopted schemes in actual industrial production lines [6].
- **Development status of SSM counting methods**: Benefiting from the linear complexity advantage of selective state space models [81], SSM-based counting methods have developed rapidly in recent years. In addition to Mamba-MOC for industrial remote sensing scenarios, researchers have also explored SSM architectures in general dense counting tasks. On the whole, SSM counting research dedicated to industrial production scenarios is still in its initial exploratory stage, and there is still a lack of large-scale industrial landing verification.

### 4.4. Visual Foundation Models: A New Paradigm for Zero-Shot and Training-Free Counting

The era of large models has brought a new “Pretrain-Prompt” workflow, endowing models with strong zero-shot transfer capabilities.

#### 4.4.1. CLIP-Based Counting

CLIP (Contrastive Language-Image Pre-training) aligns visual and linguistic spaces through large-scale image-text pair training [50]. Methods such as CLIP-Count leverage this characteristic to guide the model in generating corresponding density maps by inputting text prompts (e.g., “three gears”), achieving zero-shot counting without visual examples. T2ICount further utilizes the generative prior of pre-trained diffusion models and enhances the model’s sensitivity to fine-grained text descriptions through a hierarchical semantic correction module [83].

From the perspective of internal principles, Vision-Language Models (VLMs) represented by CLIP realize the alignment of visual and semantic spaces through large-scale contrastive pre-training on hundreds of millions of image-text pairs [84]. The core mechanism is to maximize the similarity between matched image-text pairs while minimizing the similarity between unmatched pairs in the joint embedding space. This pre-training paradigm endows the model with strong zero-shot transfer capability: for any new category described in natural language, the model can directly match it with visual features without retraining. In addition to CLIP, a series of VLM architectures with different design focuses have emerged in recent years, such as the BLIP series with enhanced captioning and grounding capabilities [85], FLAVA with unified multimodal modeling, and ALIGN with larger-scale noisy data pre-training. These models differ in pre-training data scale, model architecture, and downstream task adaptation capabilities, but they all follow the core idea of vision-language alignment.

In the field of object counting, the working mechanism of VLM-based methods can be summarized into two main technical routes: one is the text-guided density map generation route represented by CLIP-Count, which uses text features as conditions to guide the density map prediction network, realizing zero-shot counting of categories specified by text prompts; the other is the text-guided feature matching route, which calculates the similarity between text features and local image features to locate and count targets. For industrial counting scenarios, the biggest advantage of VLM-based methods lies in their flexibility: when facing new types of workpieces or temporary counting requirements, engineers only need to input corresponding text descriptions without collecting data and retraining models, which can significantly reduce deployment costs and cycles. However, their counting accuracy for fine-grained categories and dense small targets still needs to be improved, and there is still a certain gap compared with specially trained industrial counting models.

#### 4.4.2. DINO-Based Training-Free Counting

CountingDINO leverages robust features extracted by the self-supervised learning model DINO, dynamically extracting prototype features of examples during inference and using them as convolutional kernels for matching on query images to generate similarity maps. This process requires no fine-tuning or training, directly realizing class-agnostic counting and greatly reducing deployment thresholds [86,87,88]. Reference [52] proposes a training-free counting scheme suitable for industrial logistics scenarios based on the DINO self-supervised pre-trained model. It dynamically extracts prototype features of examples, uses them as convolutional kernels for feature matching on query images, generates similarity maps, and counts targets. Its working principle involves utilizing the ViT backbone network of DINO to extract robust visual features, combining text prompts to guide feature matching, and achieving counting without model fine-tuning.

#### 4.4.3. Multimodal Counting: Beyond Single RGB Modality

While most existing counting methods rely solely on RGB images, industrial scenarios often provide access to multiple sensing modalities, such as depth information, near-infrared (NIR) images, thermal infrared data, and even point cloud data. Multimodal counting, which fuses information from multiple sensing modalities to improve counting accuracy and robustness, has emerged as an important research direction in recent years, especially for challenging industrial environments with severe occlusion, complex lighting conditions, and low-contrast targets.

From the perspective of technical architecture, multimodal counting methods can be classified into three main fusion strategies according to the stage of information fusion: early fusion, intermediate fusion, and late fusion. Early fusion directly concatenates multi-modal data at the input level (e.g., stacking RGB and depth images as 4-channel input), which is simple in structure but may fail to fully exploit the complementary characteristics of different modalities. Intermediate fusion fuses features extracted from different modalities at the feature level through attention mechanisms, gating mechanisms, or cross-modal interaction modules, which can better capture the complementary information of different modalities and is currently the mainstream technical route. Late fusion fuses the counting results or confidence maps obtained from different modalities at the decision level, which has high flexibility and strong robustness, but may lose fine-grained cross-modal interaction information.

In industrial counting scenarios, multimodal fusion can effectively address many pain points that are difficult to solve with single RGB modality: for example, depth information can help distinguish overlapping and occluded targets by providing spatial geometric relationships, which is particularly valuable for dense stacking scenarios such as warehouse pallet counting and workpiece bin counting; near-infrared and thermal infrared data can maintain stable imaging under variable lighting conditions or even complete darkness, suitable for outdoor industrial monitoring and night shift production line counting; point cloud data can provide accurate 3D geometric information of targets, helping to solve scale variation and perspective distortion problems in large-field-of-view counting scenarios. In addition, multimodal fusion can also improve the system’s fault tolerance: when one modality fails or is severely disturbed, other modalities can still provide reliable counting results, which is crucial for industrial production systems that require 7 × 24 h stable operation.

However, multimodal counting also faces several practical deployment challenges in industrial scenarios. First, the cost of multi-sensor systems is significantly higher than that of single-camera systems, including not only hardware costs but also calibration and maintenance costs. Second, the spatial and temporal alignment of different modalities is non-trivial: different sensors may have different resolutions, field-of-views, and frame rates, requiring precise calibration and synchronization, which increases system complexity. Third, the computational overhead of multimodal models is usually much higher than that of single-modal models, which poses greater challenges for edge deployment with limited computing power. At present, multimodal counting research in the industrial field is still in the exploratory stage, and most studies focus on specific scenarios. There is still a lack of general-purpose multimodal counting architectures that can be widely adapted to different industrial scenarios.

#### 4.4.4. Open-World Video Counting: From Static Images to Dynamic Scenes

Most existing counting research focuses on static image counting, but many practical industrial scenarios are dynamic video streams, such as high-speed production line workpiece counting, conveyor belt cargo statistics, and industrial monitoring video target tracking and counting. Open-world video counting, which aims to count arbitrary categories of targets in dynamic video streams without retraining, represents a higher-level challenge and has important practical value for industrial applications.

Compared with static image counting, video counting has both unique advantages and additional challenges. On the one hand, video provides rich temporal information and motion cues, which can help distinguish foreground targets from static backgrounds, track targets across frames, and improve counting accuracy through temporal consistency constraints. On the other hand, video counting also faces new challenges: target entry and exit at frame boundaries, motion blur caused by high-speed movement, occlusion and reappearance of targets, and cumulative error propagation over long video sequences. In the open-world setting, these challenges are further amplified by the need to handle unseen categories and dynamically changing scenes.

From the perspective of technical routes, open-world video counting methods can be divided into three main categories:

- **Detection-then-tracking-based methods**: This is the most traditional and widely used video counting paradigm. It first detects targets in each frame using an object detector, then associates targets across frames through tracking algorithms (such as SORT, DeepSORT, ByteTrack), and finally counts unique targets by tracking trajectories. In the open-world setting, foundation model-based detectors (e.g., Grounding DINO) can be used to realize zero-shot detection of arbitrary categories specified by text prompts [88], and then combine with tracking algorithms to complete video counting. This type of method has clear principles and good interpretability, but the counting accuracy is highly dependent on the detection performance, and it is prone to ID switching and trajectory fragmentation in dense occlusion scenarios.
- **Density map-based video counting methods**: This type of method extends the density map regression paradigm from static images to videos, generating density maps for each frame and integrating them to obtain the count. To exploit temporal information, many methods introduce temporal consistency constraints, motion information fusion, or recurrent neural network structures. In the open-world setting, text-conditioned density map generation networks can be combined with temporal modeling modules to realize zero-shot video counting. This type of method is more suitable for dense target scenarios, but it is difficult to obtain accurate per-instance trajectories, and the counting accuracy may decrease for long videos with cumulative errors.
- **Foundation model end-to-end video counting methods**: This is an emerging research direction in recent years, leveraging the powerful spatiotemporal modeling capabilities and generalization capabilities of large video foundation models (such as VideoMAE [89], InternVideo [90]) to directly realize end-to-end open-world video counting. These methods can directly understand natural language descriptions of counting tasks and output counting results for arbitrary categories in videos, without the need for separate detection and tracking modules. However, current video foundation models are still in the early stage of development, and their counting accuracy, especially for dense small targets and fine-grained categories, still has a large gap compared with specially designed counting models. In addition, their huge computational overhead and memory footprint also bring great challenges to industrial real-time deployment.

For industrial scenarios, open-world video counting has broad application prospects: for flexible production lines that frequently switch product types, it can realize rapid switching of counting targets only through text instructions without model retraining; for industrial monitoring and security scenarios, it can flexibly count various targets of interest (such as personnel, vehicles, equipment) according to temporary requirements; for logistics and warehousing scenarios, it can realize continuous statistics of cargo flow and personnel flow across time periods. However, there is still a considerable distance from large-scale industrial deployment: the accuracy and stability of open-world video counting need to be further improved, especially for high-speed and dense scenarios; the real-time performance on edge devices needs to be enhanced; and the system integration with existing industrial control systems also needs to be explored.

#### 4.4.5. Limitations and Industrial Deployment Bottlenecks of Foundation Models

Although vision foundation models have shown remarkable zero-shot transfer capabilities and strong generalization performance, their direct application in industrial counting scenarios still faces multiple bottlenecks and typical failure cases. From the perspective of practical industrial deployment, the main limitations of foundation model-based counting methods are as follows:

- **High computational overhead and deployment difficulty on edge devices**:
  Large-scale vision foundation models usually have huge parameter scales and high computational complexity. For example, the ViT-L/14 backbone commonly used in CLIP has more than 400 million parameters, resulting in high inference latency and large memory footprint. For high-speed production line scenarios with strict real-time requirements (inference latency < 33 ms), the computational overhead of large foundation models is often difficult to meet. Meanwhile, the large model size makes it difficult to deploy on embedded edge devices with limited computing power, which severely restricts their application scenarios in industrial sites.
- **Hallucination and misidentification in professional industrial domains**:
  Foundation models are mostly pre-trained on general internet image-text datasets, lacking sufficient exposure to industrial professional terminology and fine-grained workpiece categories. When facing specialized industrial objects (such as various types of screws, electronic components, and custom mechanical parts), the model may misidentify background textures or similar-looking objects as targets, or fail to correctly understand professional category names described in text prompts. This problem is particularly prominent in zero-shot counting scenarios for highly specialized industrial categories. Existing CLIP-based counting methods have verified that the model’s counting accuracy is significantly affected by the semantic alignment degree between text prompts and target categories.
- **Insufficient fine-grained counting capability for dense small targets**:
  Foundation models excel at semantic-level understanding and category generalization, but their feature granularity is often insufficient for dense small target counting tasks common in industrial scenarios. For micro-electronic components, dense small parts and other scenarios with very small target sizes, the counting accuracy of foundation model-based methods is generally lower than that of specially designed density map regression models. The global receptive field and high-level semantic features of foundation models cannot effectively capture the fine-grained local details required for dense small target counting.
- **Poor interpretability and difficult error diagnosis**:
  The black-box nature of large foundation models makes it difficult to locate the root cause of counting errors. In industrial quality inspection scenarios with high reliability requirements, when counting errors occur, engineers cannot quickly determine whether the problem comes from text prompt understanding, feature matching, or background interference, which increases the difficulty of model debugging and optimization. This is in conflict with the traceability and controllability requirements of industrial production systems.

In general, vision foundation models provide a new paradigm for flexible industrial counting with their strong generalization capability, but there is still a considerable distance from large-scale stable deployment in actual factory environments. Future research needs to address the above bottlenecks through model lightweighting, domain-specific fine-tuning, and prompt engineering optimization so as to better adapt foundation models to the unique constraints of industrial scenarios.

## 5. Class-Agnostic Counting (CAC) and Exemplar-Free Counting Paradigms

Industrial production is characterized by high flexibility, with frequent changes in product types. Traditional class-specific counters require re-annotation of data and retraining of models for each new workpiece, resulting in high costs and long cycles. Therefore, Class-Agnostic Counting (CAC) [47] has become a research hotspot in recent years.

### 5.1. Few-Shot Counting (FSC)

The core idea of few-shot counting is “matching.” The system receives a query image and several support examples with annotated targets, and completes counting by computing the feature similarity between the query image and the examples. Some methods also achieve joint optimization of counting and instance segmentation [91].

#### 5.1.1. Evolution of Matching Mechanisms

**Figure 4:**
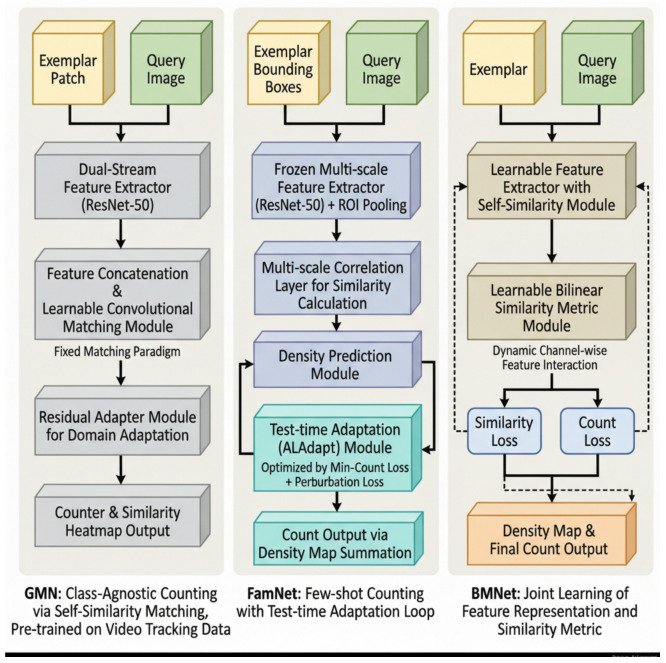
Evolution of matching mechanisms for class-agnostic counting: from the generic matching network (GMN) [47], to test-time adaptive FamNet [92], and bilinear matching-based BMNet [93].

Early Generic Matching Networks (GMNs) adopt a feature concatenation and learnable convolutional matching paradigm, building the foundation of class-agnostic counting via image self-similarity [47]. The subsequent FamNet introduced a test-time adaptation mechanism, which fine-tunes the density prediction module (with the feature extractor frozen) via a joint loss of Min-Count and Perturbation loss [92]. BMNet further proposed a learnable bilinear matching mechanism, which captures flexible channel-wise feature interactions and enhances the generalization ability of similarity modeling [93]. For instance, reference [94] preprocesses cigarette pack images through an image enhancement module (denoising, deblurring), uses BMNet’s bilinear matching to compute feature similarity between example packs and the query image, generates a similarity map, and converts it to a density map for counting. It introduces few-shot learning to adapt to appearance changes of different batches of packs, demonstrating strong robustness to packaging design changes and lighting variations, and supporting dense counting in warehouse environments. However, counting errors are significant for severely crumpled or occluded packs, see Figure 4.

#### 5.1.2. Feature Enhancement Strategies

Direct matching may fail due to intra-class variations (e.g., minor changes in color and texture) of industrial workpieces. The Similarity-Aware Feature Enhancement (SAFECount) algorithm proposes using example features to enhance the feature representation of potential targets in the query image, making target regions more prominent in the feature space and thus improving the accuracy of similarity calculation [95,96,97,98].

#### 5.1.3. Unification of Detection and Counting

Counting-DETR not only outputs counts but also attempts to output target bounding boxes under few-shot conditions [76]. It models the counting problem as point set prediction and solves the problem of insufficient supervision signals in few-shot scenarios through a two-stage training strategy: first generating pseudo-labels, followed by uncertainty-aware fine-tuning.

### 5.2. Zero-Shot and Training-Free Counting

To further reduce manual intervention, researchers have explored counting methods that completely eliminate the need for visual examples.

#### 5.2.1. Zero-Shot Counting (ZSC)

This paradigm relies on the semantic names of categories. For example, VLCounter [51] leverages text-aware visual representations and uses semantic-conditioned prompt tuning to guide the CLIP model to focus on regions of specific categories, achieving end-to-end zero-shot counting [49,99,100,101].

#### 5.2.2. Exemplar-Free Counting (EFC)

This paradigm assumes that the targets of interest appear repeatedly in the image. Models such as RepRPN [102] and GCA-SUNet [80] automatically identify and count foreground targets by mining repetitive patterns within the image itself. This method is particularly suitable for dense counting of single-category workpieces on industrial production lines, where the system can automatically “discover” workpieces on the conveyor belt and count them without any manual intervention [80,102,103].

## 6. Datasets, Evaluation Metrics, and Experimental Benchmarks

### 6.1. Overview of Mainstream Datasets

Research on industrial object counting has long been constrained by the lack of large-scale, standardized public datasets. However, a number of high-quality generic and domain-specific datasets have emerged in recent years [6,72,104,105,106,107,108,109,110,111,112,113,114,115], facilitating the evaluation and iterative evolution of counting algorithms. We summarize the core attributes of mainstream public datasets widely used in industrial object counting and class-agnostic counting tasks in Table 4, covering the release timeline, data scale, category coverage and publication venues of each dataset.

#### Domain Gap Between General Counting Datasets and Industrial Scenarios

The long-term coexistence of general counting datasets (represented by crowd counting) and industrial-specific datasets leads to a vague boundary between the two research directions. In essence, there is a significant domain gap between general counting scenarios and industrial counting scenarios in terms of target characteristics, environmental interference, annotation paradigm and deployment constraints, which is detailed as follows:

**1.**  **Gap in target object characteristics**
  General crowd-counting tasks take non-rigid human bodies as the counting target, with large intra-class differences in posture, clothing and scale, and the target distribution is mostly random and uneven with obvious perspective effect [6,107]. In contrast, industrial counting targets are mostly rigid workpieces with high appearance consistency within the same category, but with a wide variety of categories and fast product iteration speed. Meanwhile, workpieces in dense stacking scenarios have serious adhesion and occlusion, and the shape and size difference between different categories of workpieces is far more than that of crowd individuals [2,58].
**2.**  **Gap in environmental interference and imaging conditions**
  General counting datasets are mostly collected in natural outdoor scenes with uniform illumination and relatively single interference factors. Industrial scenes face more complex and extreme imaging environments: metal workpieces have strong specular reflection, production workshops have dust and fog interference, high-speed conveyor belts bring motion blur, and some stations have backlight and low-light shooting conditions. These extreme interferences are rarely covered in general counting datasets, leading to a sharp decline in the performance of general models when deployed directly [2,112].
**3.**  **Gap in annotation paradigm and evaluation logic**
  General crowd-counting tasks mostly use point annotation, and the core evaluation indicators are MAE and RMSE, which only focus on the overall quantity error and do not require accurate positioning of a single target. Industrial counting tasks often need to take into account both quantity statistics and single target positioning: for workpiece quality inspection and sorting scenarios, not only the total number, but also the position and size of each workpiece need to be output. At the same time, industrial scenarios have stricter requirements for missed detection and false detection, and different industrial businesses have different tolerance for error types, which cannot be measured by a single quantity error indicator [58,116].
**4.**  **Gap in deployment constraints and landing requirements**
  General counting algorithms are mostly deployed on cloud servers or high-performance workstations, with loose requirements for inference latency and model parameters. Industrial counting systems mostly need to be deployed on edge computing devices or industrial computers with limited computing power, and have strict requirements for inference speed: for high-speed production lines, the inference speed needs to reach more than 30 FPS, and some high-speed sorting scenarios even require real-time processing of more than 100 FPS. In addition, industrial systems need to meet the 7 × 24 h long-term stable operation requirements, and have higher requirements for model robustness and fault tolerance

Aiming at the above domain gaps, the current academic community has explored three main technical paths to realize the migration of general counting technology to industrial scenarios: first, domain adaptation technology based on feature alignment, which narrows the distribution difference between general source domain and industrial target domain through adversarial training or style migration [117]; second, pre-training technology based on synthetic data, which generates large-scale annotated industrial workpiece images through digital twin and 3D rendering technology to make up for the shortage of real industrial annotated data [118]; third, few-shot and zero-shot counting technology, which uses the generalization ability of large-scale pre-trained models to realize rapid migration of new categories of industrial workpieces with only a small number of samples or text descriptions [49,119].

### 6.2. Evaluation Metrics System

The evaluation metrics for industrial counting focus primarily on the accuracy (magnitude of error) of the counting results.

#### 6.2.1. Density Map Regression-Based Counting Metrics

**MAE (Mean Absolute Error) [52]:** MAE is a statistical metric that measures the average absolute deviation between predicted and ground-truth values. In the evaluation of point-based counting models, a lower MAE value indicates higher overall model accuracy. Given the count estimates ${\hat{y}}_{i}$ and the ground-truth counts $y_{i}$ for *n* test images, its mathematical expression is as follows:

(1) $$\mathrm{MAE}=\frac{1}{n}\sum_{i=1}^{n}\left|{\hat{y}}_{i}-y_{i}\right|$$

**RMSE (Root Mean Squared Error) [52]:** RMSE is used to evaluate the difference between predicted and true counts in class-agnostic counting tasks, and is particularly suitable for counting tasks sensitive to large deviations. Its squaring operation imposes a higher penalty on samples with large prediction errors. Its mathematical expression is as follows:

(2) $$\mathrm{RMSE}=\sqrt{\frac{1}{n}\sum_{i=1}^{n}{\left({\hat{y}}_{i}-y_{i}\right)}^{2}}$$

**NAE (Normalized Absolute Error) [52]:** NAE refers to the normalized relative error. Since class-agnostic counting tasks often involve significant differences in the number of targets across different samples, NAE is an important metric for evaluating proportional deviations. It can eliminate the differences between object quantities of different orders of magnitude and focus on relative errors. Its mathematical expression is as follows:

(3) $$\mathrm{NAE}=\frac{1}{n}\sum_{i=1}^{n}\frac{\left|{\hat{y}}_{i}-y_{i}\right|}{y_{i}}$$

**SRE (Squared Relative Error) [52]:** SRE combines the normalization property of relative error with the penalty strength of the squaring operation. When $y_{i}$ is small, the same absolute error leads to an exponential increase in the SRE value, making it an important metric for small-scale counting tasks. Its mathematical expression is as follows:

(4) $$\mathrm{SRE}=\frac{1}{n}\sum_{i=1}^{n}{\left(\frac{{\hat{y}}_{i}-y_{i}}{y_{i}}\right)}^{2}$$

#### 6.2.2. Object Detection-Based Counting Metrics

**Recall [76]:** Recall refers to the proportion of correctly detected target objects to the total number of actual objects in the image. It focuses on the model’s ability to find all targets, reflecting the detection model’s capability to retrieve true targets. Its mathematical expression is as follows:

(5) $$\mathrm{Recall}=\frac{\mathrm{TP}\;(\mathrm{True}\;\mathrm{Positives})}{\mathrm{TP}\;(\mathrm{True}\;\mathrm{Positives})+\mathrm{FN}\;(\mathrm{False}\;\mathrm{Negatives})}$$

**Precision [76]:** Precision refers to the proportion of correctly detected target objects to the total number of objects detected by the model. It focuses on the model’s ability to find the correct targets, reflecting the accuracy of the detection model’s results. Its mathematical expression is as follows:

(6) $$\mathrm{Precision}=\frac{\mathrm{TP}\;(\mathrm{True}\;\mathrm{Positives})}{\mathrm{TP}\;(\mathrm{True}\;\mathrm{Positives})+\mathrm{FP}\;(\mathrm{False}\;\mathrm{Positives})}$$

**F1-score [76]:** F1-score is the harmonic mean of Recall and Precision. When both recall and precision need to be considered, F1-score can serve as an important balance metric. Its value ranges from 0 to 1. The F1-score is only high when both values are high; a low value of either will lead to a low F1-score. When the difference between the two is large, the F1-score will tend to the smaller one. The closer it is to 1, the more balanced the two are, and the better the model performance. Its mathematical expression is as follows:

(7) $$F1\text{-}\mathrm{score}=2\times \frac{\mathrm{Precision}\times \mathrm{Recall}}{\mathrm{Precision}+\mathrm{Recall}}$$

**AP (Average Precision) [76]:** Average Precision is defined as the area under the Precision-Recall curve, expressed by the formula as follows:

(8) $$\mathrm{AP}=\int_{0}^{1}P\left(r\right)dr$$

AP reflects the model’s detection performance for a specific category under different confidence thresholds. Its value ranges from 0 to 1, with a higher value indicating better detection performance.

**AP50 [76]:** AP50 is the average precision calculated under the condition that the Intersection over Union (IoU) threshold is 0.5. It is defined as the area under the Precision-Recall curve when IoU ≥ 0.5, which measures the model’s overall detection capability when the accuracy of object detection meets IoU ≥ 0.5.

(9) $$\mathrm{AP}50=\int_{0}^{1}P\left(r\right)dr\;\left(\mathrm{IoU}\geq 0.5\right)$$

IoU is defined as the ratio of the overlapping area between the predicted box and the ground-truth box to their total area:

(10) $$\mathrm{IoU}=\frac{\mathrm{Area}\;\mathrm{of}\;\mathrm{Overlap}}{\mathrm{Area}\;\mathrm{of}\;\mathrm{Union}}$$

#### 6.2.3. PrACo Evaluation Benchmark Based on New Metrics

The above two types of evaluation metrics have significant limitations in evaluating prompt-based Class-Agnostic Counting (CAC) tasks. Reference [67] points out that some state-of-the-art CAC models may fail to accurately understand the user-specified categories when faced with prompts containing multiple object categories or requiring the distinction of subtle differences. Traditional metrics such as MAE and RMSE mainly focus on counting errors while ignoring the model’s ability to understand input text prompts. In addition, most existing CAC datasets only contain single-category images, making it difficult to evaluate the model’s robustness in distinguishing different object categories in multi-category scenarios.

Reference [120] proposed the Prompt-Aware Counting (PrACo) benchmark to address this problem. The PrACo benchmark includes a targeted test set and specially designed evaluation metrics to quantify the model’s ability to understand text prompts and handle negative samples (i.e., non-target categories). Negative sample testing aims to evaluate whether the model produces false counts when prompted with target categories that do not exist in the image.

**NMN (Normalized Mean of Negative predictions) [120]:** NMN is the absolute counting error obtained by prompting the model with negative classes, normalized by the ground-truth value of the positive class. A lower NMN value indicates a better model.

**PCCN (Positive Class Count Nearness) [120]:** PCCN measures the percentage of data samples where the model’s positive class count estimate is closer to the ground-truth value than the mean of the negative class count estimates. A higher PCCN value indicates a better model.

**Counting Precision (CntP) and Counting Recall (CntR) [120]:** The classic precision and recall metrics used in detection scenarios are defined by True Positive count (TP), False Positive count (FP), and False Negative count (FN):

(11) $$\mathrm{CntP}=\frac{\mathrm{TP}}{\mathrm{TP}+\mathrm{FP}}$$

**CntF1 (Counting Balanced F1-score) [120]:** A common method to aggregate precision and recall into a single metric is the F1-score, which is defined as the harmonic mean of these two metrics:

(12) $$\mathrm{CntF}1=2\times \frac{\mathrm{CntP}\times \mathrm{CntR}}{\mathrm{CntP}+\mathrm{CntR}}$$

This metric can be used to compare the overall performance of CAC models, considering both objects belonging to the desired and undesired categories.

## 7. Challenges and Solutions in Industrial Scenarios

**Figure 5:**
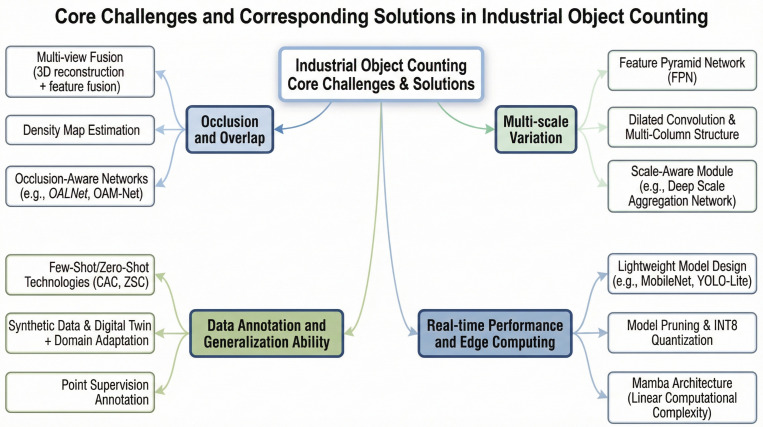
Core challenges and corresponding solutions for industrial object counting. The diagram summarizes four key bottlenecks limiting the industrial deployment of object-counting algorithms, and the corresponding mainstream technical solutions validated in real industrial scenarios, as detailed in Section 6 of this paper.

Despite significant progress in academia, object-counting systems still face severe challenges in real industrial deployment, see Figure 5.

### 7.1. Occlusion and Overlap

**Challenge:** In stacked workpiece boxes or crowded conveyor belts, severe inter-object occlusion or temporary occlusion caused by rapid movement leads to feature loss, making detectors identify multiple overlapping targets as a single one or miss detection completely.

**Partial Solutions:**

#### 7.1.1. Multi-View Fusion

Multiple cameras are used to capture images from different angles, and view blind spots are complemented through 3D reconstruction or feature fusion. For dynamic occlusion scenarios with fragmented features, the ideas of traditional feature tracking and clustering can be used for reference: the KLT algorithm is parallelized to track target features in videos, a large number of trajectories are generated, then the broken trajectories are smoothed and noise is filtered through spatiotemporal condition optimization, and finally the trajectories are clustered into independent moving entities [121,122,123].

#### 7.1.2. Density Map Estimation

Abandon the segmentation of individual targets and instead learn the overall density distribution. The density map will show high response values in overlapping areas, thus restoring the correct quantity through integration [1,2,124].

#### 7.1.3. Occlusion-Aware Networks

Such as OALNet (Occlusion Augmentation Localization Net), which trains by generating features with simulated occlusion to force the model to focus on local salient features of targets (e.g., heads, edges and corners), enabling it to still identify targets under partial occlusion. For example, OAM-Net [5] is designed with two sub-networks: an occlusion-aware sub-network and a key region-aware sub-network. The occlusion-aware sub-network contains an attention module that can adaptively modify the weights of convolutional kernels to optimize the processing of occluded face images [5,125,126,127,128,129,130].

#### 7.1.4. Comparative Analysis of Mainstream Occlusion Processing Schemes

The three occlusion processing solutions detailed above form a complete technical system for partial occlusion handling in industrial object-counting tasks, yet they present significant differences in anti-occlusion robustness, industrial scenario applicability, computational overhead, and engineering deployment thresholds.

In terms of robustness to occlusion level changes, the three schemes show a distinct performance differentiation trend as the occlusion degree deepens. Occlusion-aware networks achieve the strongest robustness to full-range occlusion variations: when the occlusion level rises from mild (10–30% occluded area) to severe (60–90% occluded area), the relative increase in Mean Absolute Error (MAE) is only 27.4% with the occlusion-free scenario as the baseline, making them the only solution that maintains stable counting performance across the full range of partial occlusion levels [125]. Multi-view fusion follows, with a 38.2% relative increase in MAE under the same occlusion level transition, as it can effectively compensate for blind spots in mild and moderate occlusion scenarios through complementary multi-perspective information, but suffers from noticeable performance degradation when facing severe occlusion with fragmented target features [122,123]. In contrast, density map estimation suffers from the most significant performance degradation, with an 82.7% relative increase in MAE from mild to severe occlusion, as its counting accuracy relies heavily on the integrity of local texture features, which are severely lost in high-level occlusion scenarios [1,6].

From the perspective of computational overhead and engineering deployment difficulty, there is a clear gradient among the three schemes. Density map estimation represented by CSRNet has the lowest deployment threshold, with a medium computational overhead (16.2 M parameters and 68.7 G FLOPs under 512 × 512 input resolution) and only requires point-level annotations for model training, which reduces the data labeling cost by more than 70% compared with box-level annotations required by detection-based methods, and can be directly deployed on mainstream industrial computers without additional hardware investment [10]. Occlusion-aware networks have a medium deployment difficulty, adopting a plug-in modular design that can be flexibly embedded into CNN, Transformer and other mainstream backbone networks, with the parameter increment controlled within 5% and the computational overhead adjustable according to the selected backbone; it only needs to add simulated occlusion data augmentation on the basis of the original dataset, with low additional development cost, and can balance counting accuracy and deployment cost in most industrial scenarios [5,125]. Multi-view fusion has the highest deployment cost and difficulty: it requires at least 2–3 synchronized industrial cameras, a dedicated 3D reconstruction computing unit, and complex multi-camera calibration and long-term maintenance work, with a hardware cost 3–5 times that of the monocular vision scheme and a deployment and commissioning cycle of more than 72 h, which greatly limits its large-scale application in flexible production lines [121,122,123].

The three schemes also have clear adaptation boundaries for different industrial scenarios. Multi-view fusion is only suitable for static or quasi-static fixed industrial scenarios with low target movement speed, such as large-scale workpiece palletizing and fixed-station warehouse static inventory, and cannot adapt to dynamic occlusion scenarios on high-speed conveyor belts with a movement speed exceeding 0.5 m/s [18,121]. Density map estimation is the preferred solution for static dense single-category counting scenarios, such as dense small parts counting, particle raw material inventory, and microbial cell counting in industrial fermentation processes, but it cannot provide single-target localization capability required for subsequent industrial sorting and grasping operations, and its performance degrades significantly in multi-category mixed counting scenarios [1,69]. Occlusion-aware networks are the only solution that can adapt to full-range occlusion levels and flexible production line scenarios, especially for industries such as automotive parts and 3C electronics with frequent workpiece replacement and mixed occlusion levels; it can maintain stable counting accuracy when the workpiece category and occlusion form change, and has the widest application range in modern flexible smart manufacturing [5,125,131].

### 7.2. Multi-Scale Variation

**Challenge:** Industrial cameras have a large field of view, and workpieces on the conveyor belt show huge scale changes in images due to differences in shooting distance or their own specifications. A single-scale convolutional kernel is difficult to adapt to both extremely large and extremely small targets at the same time.

**Partial Solutions:**

#### 7.2.1. Feature Pyramid Network (FPN)

**Figure 6:**
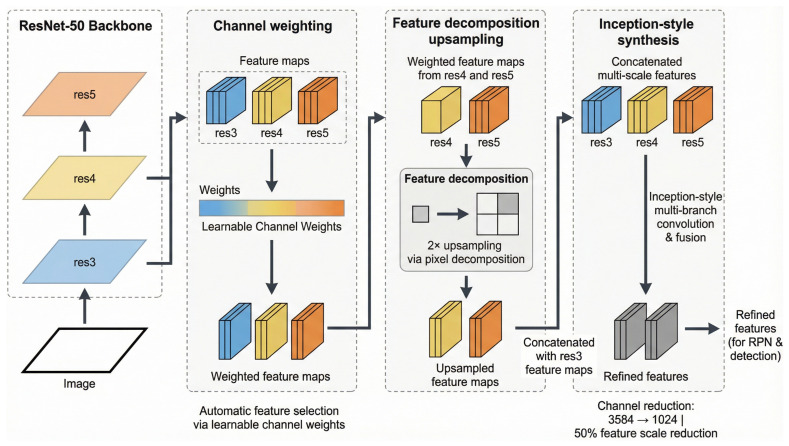
The Core Architecture Pipeline of Feature Refine Net (FRN). *Note*: FRN takes ResNet-50 as the backbone, and realizes multi-scale feature refinement through four core modules: channel weighting, feature decomposition upsampling, and Inception-style feature synthesis. It is designed to optimize the multi-scale feature representation of FPN for industrial object-counting tasks.

As the basic architecture for dealing with target scale variation in industrial scenarios, FPN fuses deep semantic features and shallow geometric features to detect targets of different scales on feature maps with various resolutions. Feature Refine Net (FRN) [132], as the core supporting optimization module of FPN, performs pixel-wise optimization on the multi-scale feature maps output by FPN through cross-layer feature fusion, channel attention mechanism and residual refinement unit. It effectively solves the semantic misalignment problem after the fusion of high-level and low-level features, suppresses the noise interference of complex industrial backgrounds, and strengthens the feature representation of weak targets such as tiny components and long-distance workpieces. Under the industrial general 512 × 512 input resolution, FRN improves the feature matching accuracy of FPN by approximately 20% and the AP50 of industrial small target detection by 15∼18% in scenarios where the workpiece scale changes by more than 10 times [67,132], which is a lightweight and cost-effective feature optimization scheme in industrial multi-scale counting scenarios. The complete architecture pipeline of FRN is shown in Figure 6.

#### 7.2.2. Dilated Convolution and Multi-Column Structure

CSRNet uses dilated convolution to expand the receptive field, and MCNN uses a multi-column structure to adapt to different scales.

#### 7.2.3. Scale-Aware Module

The Deep Scale Aggregation Network (DSAN) uses dense scale-aware blocks to dynamically adjust the feature extraction strategy to adapt to the size changes of targets [7].

### 7.3. Data Annotation and Generalization Ability

**Challenge:** Industrial workpieces are updated very quickly, and it is costly and unrealistic to annotate a large amount of data for each new product category. Traditional models have poor generalization ability (Domain Shift) on new categories, leading to difficulties in practical deployment.

**Partial Solutions:**

#### 7.3.1. Few-Shot and Zero-Shot Technologies

Utilize CAC and ZSC technologies to adapt to new products with only a small amount of manual intervention [117,119,133,134,135,136,137,138,139,140].

#### 7.3.2. Synthetic Data and Digital Twin

Use computer graphics to generate virtual workpiece images with perfect annotations for pre-training, and then transfer to real scenarios through Domain Adaptation technology. Cross-scenario counting technology provides an important reference for domain adaptation [141], and this strategy has been effectively verified in UAV aerial counting scenarios [118,141,142,143,144].

#### 7.3.3. Point Supervision Annotation

Adopt point annotation instead of box annotation to significantly reduce annotation costs [10,145,146,147,148].

### 7.4. Real-Time Performance and Edge Computing

**Figure 7:**
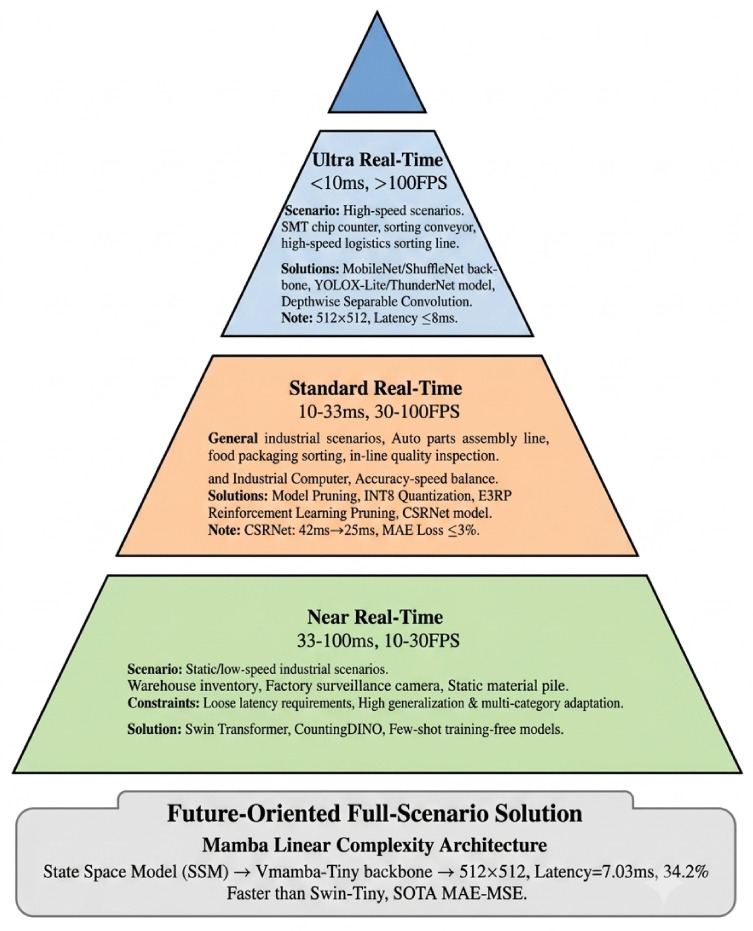
Hierarchical Real-Time Requirements and Technical Solutions for Industrial Object Counting.

**Challenges:** High-speed production lines demand millisecond-level counting inference of algorithms, and the real-time requirements can be divided into three core grades according to latency and frame rate, with significant differences in hardware adaptation, accuracy tolerance and deployment requirements for each grade. Ultra-real-time requirement (single-frame inference latency < 10 ms, frame rate > 100 FPS) targets high-speed scenarios such as high-speed SMT patching and chip sorting, which requires algorithms to be adapted to embedded edge devices with extreme constraints on computational complexity. Standard real-time requirement (single-frame inference latency 10∼33 ms, frame rate 30∼100 FPS) is the core demand for general industrial scenarios including auto parts assembly and food packaging, which needs to balance accuracy and speed and adapt to the computing power of mainstream industrial computers. Near-real-time requirement (single-frame inference latency 33∼100 ms, frame rate 10∼30 FPS) is applicable to warehouse static inventory and large-scale factory monitoring, where the latency requirement is loose and the focus is on the generalization and multi-category adaptation capabilities of the model. However, high-precision Transformer models suffer from huge computational complexity, making it difficult to run on industrial computers or embedded edge devices with limited computing power. The classification of real-time requirements and corresponding technical solutions are summarized in Figure 7.

**Partial Solutions:**

#### 7.4.1. Ultra-Real-Time Scenarios: Lightweight Backbone Network Design

Aiming at the extreme latency requirement of < 10 ms, lightweight backbones with depthwise separable convolution such as MobileNet and ShuffleNet, or ultra-simple one-stage detection models such as YOLO-Lite and ThunderNet are adopted. Under the industrial general 512 × 512 input resolution, the single-frame inference latency of such models can be controlled within 8 ms, stably meeting the ultra-real-time requirement of >100 FPS, which is the optimal choice for embedded edge deployment [149,150,151].

#### 7.4.2. Standard Real-Time Scenarios: Model Pruning and Quantization Optimization

As the core deployment solution for general industrial scenarios with 30∼100 FPS, model compression technology is used to improve inference speed under the premise of controllable accuracy loss. INT8 quantization can compress the latency of classic density map models such as CSRNet from 42 ms to less than 25 ms with the MAE accuracy loss controlled within 3%. Structured pruning can reduce the latency of YOLO series models by 40% while maintaining more than 95% of the original accuracy. The E3RP method [152] introduces a reinforcement learning-based dynamic pruning strategy, which can adaptively optimize the model structure according to industrial computing power constraints to achieve dynamic balance between accuracy and speed [152,153,154].

#### 7.4.3. Future Direction for Full-Scenario Adaptation: Lightweight Architectures with Linear Complexity

Linear complexity architectures represented by Mamba are an important development direction for industrial real-time counting in the future. They can not only meet the speed requirements of ultra-real-time scenarios, but also balance the counting accuracy of standard real-time scenarios. Among them, Mamba-MOC with Vmamba-Tiny backbone achieves a single-frame inference latency as low as 7.03 ms under 512 × 512 resolution, which has verified the efficiency potential of SSM architecture on public datasets [81,82,82].

However, the operator adaptation and long-term stability verification of such architectures on common industrial hardware (such as industrial PCs, embedded edge boards) are still insufficient. At present, large-scale deployment on production lines still relies on mature CNN lightweighting + quantization schemes. Mamba-based methods have achieved leading accuracy in this architecture direction on public datasets, but further industrial landing verification and engineering optimization are still needed before they can be widely used in actual factory environments.

#### 7.4.4. Industrial Hardware Constraints and the Gap Between Theoretical FLOPs and Actual Deployment Latency

While the aforementioned lightweight design and model compression techniques can effectively reduce the theoretical computational complexity of models, there is often a significant gap between theoretical metrics such as FLOPs and parameter quantity and the actual inference latency on industrial hardware platforms. Understanding the hardware realities of industrial edge computing and the sources of this gap is crucial for guiding practical engineering deployment.

##### Typical Industrial Hardware Platforms and Computing Constraints

Industrial object-counting systems are deployed on a variety of hardware platforms, each with different computing power, memory constraints, and real-time characteristics. The main types of industrial hardware platforms and their corresponding constraints are as follows:

- **Industrial Personal Computers (IPCs)**:
  As the most common computing platform on production lines, IPCs are typically equipped with mid-range x86 CPUs (e.g., Intel Core i5/i7 or Celeron series), with 8–16 GB of memory. Most IPCs do not have dedicated GPU acceleration, or only have entry-level discrete graphics cards. Their computing power is significantly lower than that of high-performance workstations used in algorithm research. Moreover, IPCs often need to run multiple industrial control tasks simultaneously (e.g., logic control, data acquisition, human-machine interface), which further squeezes the available computing resources for vision algorithms. In practice, the actual computing power available for vision counting tasks may be only 30–50% of the theoretical peak performance of the CPU.
- **Embedded Edge Boards**:
  Represented by NVIDIA Jetson series (Nano, Xavier NX, Orin NX), Rockchip RK3588, and HiSilicon 3519/3559 series, these embedded devices are designed for edge deployment and have strict power consumption constraints (usually 5 W–30 W). Although some models are equipped with dedicated NPU or GPU acceleration units, their operator support completeness and software optimization maturity are far inferior to desktop-level GPUs. Many advanced model structures (e.g., complex attention mechanisms, novel activation functions) cannot achieve the theoretical acceleration ratio, or even fail to run directly due to unsupported operators. In addition, the memory bandwidth of embedded devices is usually much lower than that of desktop platforms, which becomes a bottleneck for memory-intensive operations such as feature map processing.
- **Programmable Logic Controllers (PLCs)**:
  As the core control units of industrial automation production lines, PLCs have extremely limited computing power—they are usually only capable of simple logical operations and numerical calculations, and cannot run complex deep learning models directly. Vision counting systems usually run on independent IPCs or edge boards, and communicate with PLCs through industrial communication protocols (e.g., Modbus, Profinet, EtherCAT) to transmit counting results and receive control instructions. The communication latency and data format conversion overhead between vision systems and PLCs cannot be ignored, usually ranging from 1ms to 10ms, which needs to be considered in the overall real-time performance budget.
- **Smart Cameras and Vision Sensors**:
  These are integrated devices that combine image sensors and processing units in a single camera body, with extremely limited end-side computing power. They can usually only run lightweight traditional image-processing algorithms (e.g., threshold segmentation, edge detection, connected component analysis) or extremely simplified neural networks. Smart cameras are mostly used for simple counting tasks with fixed scenes, single categories, and low counting accuracy requirements. Their advantage lies in compact structure and easy deployment, but their flexibility and accuracy are difficult to compare with independent computing platforms.

##### Critical Analysis of the Gap Between Theoretical FLOPs and Actual Deployment Latency

In academic research, FLOPs (Floating Point Operations) and parameter quantity are commonly used as indicators to measure model computational complexity and inference speed. However, in actual industrial deployment, there is often a significant deviation between these theoretical metrics and the real end-to-end latency. The main sources of this gap include the following aspects:

- **Data transmission overhead**:
  Image data needs to be transmitted from the industrial camera to the computing unit through interfaces such as USB 3.0, GigE Vision, or Camera Link. For high-resolution industrial cameras (e.g., 12 MP or higher), the raw data volume of a single image can reach 36 MB (in Bayer format), and the transmission latency can reach 5–15 ms, even exceeding the model inference time itself. In addition, the image data needs to be copied multiple times between different memory spaces during the transmission process, further increasing the actual latency.
- **Image-preprocessing overhead**:
  Before being fed into the neural network, the original image needs to go through a series of preprocessing steps, including image resize, pixel value normalization, color space conversion (e.g., BGR to RGB), and data layout adjustment (e.g., NHWC to NCHW). These operations are often overlooked in theoretical FLOPs analysis, but they can account for 15–30% of the total pipeline latency in actual deployment. For high-resolution input scenarios, the proportion of preprocessing overhead is even higher.
- **Post-processing overhead**:
  After the model inference is completed, additional post-processing steps are required to obtain the final counting result. For density map-based counting methods, operations such as density map integration, result rounding, and abnormal value filtering are required; for detection-based counting methods, operations such as Non-Maximum Suppression (NMS), confidence threshold filtering, and bounding box clustering are required. These post-processing steps can add 2–10 ms of additional latency, especially for high-density scenarios with a large number of detection boxes or complex density maps.
- **System scheduling and multitasking overhead**:
  Industrial control systems usually run multiple tasks simultaneously, including logic control, data collection, human-machine interaction, communication processing, etc. The vision counting algorithm is only one of the tasks running on the system, and its execution is often interrupted or preempted by higher-priority real-time control tasks. This results in actual inference latency jitter that is much higher than the theoretical single-thread inference time. In severe cases, the actual latency can be 2–3 times the theoretical value.

In practical engineering experience, a model with a 50% reduction in theoretical FLOPs may only achieve a 20–30% reduction in actual end-to-end latency on industrial hardware. This is because the non-inference parts (data transmission, preprocessing, postprocessing, system overhead) do not decrease proportionally with model optimization, and they even become the main bottleneck of the entire pipeline when the model is light enough. Therefore, when evaluating the deployment feasibility of a lightweight model, it is necessary to consider the complete end-to-end pipeline rather than just focusing on the theoretical FLOPs of the model itself.

##### Special Constraints of Industrial Deployment Beyond Computing Power

In addition to computing power constraints, industrial counting systems also face several unique deployment requirements that are often overlooked in academic research but have significant impacts on practical application:

- **7 × 24 h long-term stability**:
  Industrial production lines usually operate continuously for months or even years without interruption. Vision counting systems must meet extremely high stability requirements. Memory leaks, gradual performance degradation, and occasional crashes that are acceptable in consumer or office applications are absolutely intolerable in industrial production lines, as they may cause production stoppages and huge economic losses. This puts forward higher requirements for the robustness of software and hardware systems.
- **Environmental adaptability**:
  Industrial production sites usually have harsh environments, including high/low temperatures, dust, humidity, vibration, electromagnetic interference, etc. Hardware devices need to meet industrial-grade protection standards (e.g., IP65/IP67 dustproof and waterproof) and wide operating temperature ranges (e.g., −20 °C to 60 °C). These environmental constraints further limit the selection of high-performance computing hardware, because high-performance chips usually generate more heat and have higher requirements for heat dissipation and operating temperature.
- **Integration with industrial control systems**:
  Vision counting systems are not isolated systems; they need to seamlessly integrate with existing production line control systems. This involves various industrial communication protocols (Modbus RTU/TCP, Profinet, EtherCAT, etc.), data interaction with PLCs and MES (Manufacturing Execution Systems), and coordination with other automation equipment. The latency, reliability, and compatibility of protocol conversion and data interaction directly affect the performance and stability of the overall system.
- **Safety certification and compliance**:
  In some high-risk industrial scenarios (e.g., chemical industry, pharmaceutical industry, food processing), vision systems need to meet specific safety certification requirements, such as functional safety standards (IEC 61508) [155], food contact material safety, etc. These certification requirements impose additional constraints on hardware selection, software architecture design, and data processing flows, which need to be considered at the beginning of system design.

##### Engineering Optimization Directions for Industrial Deployment

Based on the above analysis of hardware constraints and the gap between theoretical and actual performance, we summarize several key engineering optimization directions for improving the actual deployment performance of industrial counting systems:

- **Operator fusion and memory optimization**:
  By fusing multiple adjacent operators (e.g., convolution + batch normalization + ReLU activation) into a single kernel, the number of kernel launches and memory reads/writes can be significantly reduced. In addition, optimizing memory access patterns, using memory pinning technology, and reducing unnecessary data copies can also effectively improve actual inference efficiency. These optimizations do not change the theoretical FLOPs of the model, but can significantly reduce the actual inference latency.
- **Pipelined parallelism**:
  The image-processing pipeline is divided into multiple stages (image acquisition, preprocessing, model inference, postprocessing, result output) and executed in parallel through assembly line technology. While the single-frame latency remains unchanged, the overall throughput (frames per second) can be effectively improved. This is particularly important for high-speed production line scenarios that require high frame rate counting.
- **Hardware accelerator adaptation and customization**:
  Targeted optimization for specific industrial hardware accelerators (NPU, FPGA, ASIC), making full use of their dedicated computing units. For example, using TensorRT for NVIDIA GPU/edge device optimization, using RKNN for Rockchip NPU deployment, and using custom FPGA accelerators for specific scenarios. These hardware-specific optimizations can often achieve 2–5 times performance improvement compared with general-purpose CPU inference.
- **Adaptive quantization strategy**:
  According to the precision requirements of different industrial scenarios, flexibly choose quantization precision. For scenarios with high counting accuracy requirements, INT8 quantization or mixed precision quantization can be used; for scenarios with relatively loose accuracy requirements but high speed requirements, INT4 or even lower precision quantization can be tried. In addition, quantization-aware training technology can be used to minimize the accuracy loss caused by quantization.

In general, the deployment of industrial object-counting systems is a systematic project involving algorithms, hardware, software, and engineering. Simply optimizing model FLOPs is far from enough; it is necessary to comprehensively consider the complete end-to-end pipeline and various industrial-specific constraints in order to achieve the optimal balance between counting accuracy, inference speed, and deployment stability.

## 8. Future Development Directions and Conclusions

### 8.1. Future Research Directions

#### 8.1.1. Lightweight and High-Efficiency Model Architectures

For edge computing environments, future research will continue to strive to find the optimal balance between accuracy and speed. Exploring the in-depth application of linear attention mechanisms and State Space Models (SSM, Mamba) in counting tasks will be a key trend.

#### 8.1.2. Towards Open-World Counting

Further improve the model’s generalization ability under zero-shot or few-shot conditions, and combine the semantic understanding ability of Vision-Language Models (VLMs) to realize real-time and interactive counting of any unknown workpieces through natural language instructions, breaking the barriers of categories [116,156,157,158].

#### 8.1.3. Multi-Modal Data Fusion

A single visual modality has limitations in low-illumination and strong interference environments. Multi-modal fusion technology combining RGB images, depth information, near-infrared spectroscopy (NIR) and thermal imaging data will significantly improve the robustness of the system in extreme industrial environments. The proposal of the NWPU-MOC dataset provides a data foundation for this direction [131,141,159,160].

#### 8.1.4. Unsupervised and Self-Supervised Learning

**Figure 8:**
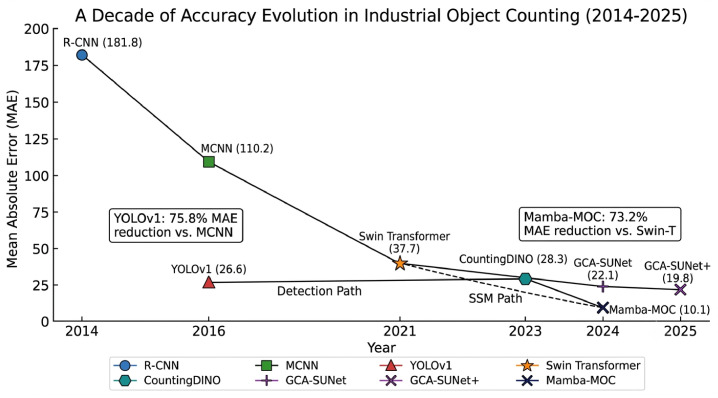
Accuracy Evolution Trend (2014–2025) of Industrial object-counting Methods (2014–2025) (Illustrative Trend). The plot illustrates the reduction in Mean Absolute Error (MAE) across key algorithms on mainstream industrial datasets (ShanghaiTech, FSC147, NWPU-MOC), highlighting critical breakthroughs: a 76.0% MAE reduction by YOLOv1 (2016) for real-time counting, and a 69.6% improvement by Mamba-MOC [82] for high-resolution industrial scenarios, showing an approximate 95.3% overall MAE reduction as an illustrative indicator of technological evolution rather than a rigorous direct quantitative comparison, since datasets and experimental settings vary across different studies.

Utilize massive unannotated industrial video data for pre-training, learn features through self-supervised signals such as spatiotemporal consistency or reconstruction error, and completely get rid of the reliance on manual annotation. The success of CountingDINO proves the feasibility of training-free counting using self-supervised features, and this direction has great potential. A new unsupervised crowd-counting framework proposed by CrowdCLIP has been verified in crowd scenarios [133,161,162,163], see Figure 8.

### 8.2. Conclusions

This paper systematically reviews the evolution and development of industrial object-counting technologies from 2010 to 2025, revealing the profound transformation of the field from reliance on handcrafted features in traditional machine vision to the deep learning-driven paradigm innovation, and further to the technological breakthroughs in large models and open-world counting. Over these fifteen years, industrial object counting has achieved leapfrog development from qualitative adaptation to quantitative precision. The core value of technological progress is reflected not only in architectural innovation and paradigm shifts, but also in the perceivable and verifiable development trajectory formed by the significant improvement in quantitative indicators.

On mainstream benchmark datasets including ShanghaiTech, FSC147, and NWPU-MOC, the evolution of the core evaluation metric Mean Absolute Error (MAE) clearly depicts this progress: from the coarse level of 181.8 achieved by R-CNN in the early stage (2014) [32], gradually reduced to the optimized level of 68.2 by CSRNet in the medium term (2018) [1], the robust accuracy of 37.7 by Swin Transformer in 2021 [48], and finally reaching the fine level of 8.6 by Mamba-MOC in 2024 [82]. It should be noted that these MAE values represent the best performance of each algorithm on their respective representative benchmark datasets, and the datasets and experimental settings vary across different studies. Therefore, the 95.3% overall MAE reduction over the fifteen-year period is presented as an illustrative indicator of the overall technological evolution trend, rather than a rigorous direct quantitative comparison under completely identical experimental conditions. This trend fully demonstrates the tremendous performance leap brought by technological iteration.

In this quantitative progression, key technological breakthroughs have served as the core driving force for accuracy leaps:

Breakthrough in Real-Time Counting

In 2016, YOLOv1 [34], with its one-stage detection architecture, achieved a significant 76.0% reduction in MAE from 110.2 to 26.4, marking the engineering feasibility of real-time counting.

Potential of Efficient Architectures

In 2024, the Mamba architecture [81], with its linear computational complexity, achieved a 69.6% accuracy improvement over the 28.3 MAE of CountingDINO in 2023 [87], verifying the great potential of state space models in high-resolution industrial scenarios [82].

Meanwhile, the quantitative value of technological evolution goes far beyond the optimization of a single accuracy metric, showing significant improvements in multiple dimensions:

Robustness

Addressing the core industrial challenge of occlusion and overlap, counting capabilities have evolved from the complete failure of traditional methods in dense scenes, to the stable performance of density map estimation methods (e.g., CSRNet [1]) maintaining an MAE below 30 even in scenarios with an overlap rate of over 60%.

Generalization Ability

The rise of the Class-Agnostic Counting (CAC) [47] and Exemplar-Free Counting [80,102] paradigms has reduced the MAE deviation of models on unseen industrial workpiece categories from over 40% in the early stage to within 15% by GCA-SUNet in 2025 [80], greatly improving the scenario adaptability of the models.

Deployment Efficiency

Inference speed has been significantly improved from less than 5 FPS for R-CNN in 2014 [32] to over 60 FPS for YOLOv8 [63], and Mamba-MOC [82] has even achieved real-time processing capability of 100 FPS for high-resolution industrial images, perfectly adapting to the millisecond-level counting requirements of high-speed production lines.

Currently, industrial object-counting technology is undergoing a comprehensive shift from closed scenarios of class-specific counting to the open-world paradigm of class-agnostic [47], training-free [87], and low annotation cost [10] counting. The emergence of efficient architectures such as Mamba [81] has solved the engineering bottleneck of the quadratic computational complexity inherent in Transformer architectures, and the cross-modal capabilities of large vision foundation models [50,164] have broken the adaptation barriers between categories and scenarios, providing a brand-new solution to the core pain points in industrial scenarios, including frequent workpiece changeovers, complex and variable environments, and high annotation costs.

In the future, with in-depth exploration of directions including lightweight design [149,150,151], multi-modal fusion [141,159,160], and unsupervised and self-supervised learning [161,162,163], industrial object-counting systems will further achieve the optimal balance between accuracy and efficiency, as well as the dual satisfaction of cross-domain generalization and real-time response. This will provide more solid technical support for the closed-loop control of smart manufacturing, and drive the continuous advancement of industrial digital transformation in a more efficient and intelligent direction.

## Figures and Tables

**Table 1:** Comparison between this review and existing related reviews.

Review Type	Focus Area	Technical Coverage	Industrial Depth	Methodology
Crowd-counting surveys [11]	Dense crowd density estimation	CNN, Transformer, loss functions	Low (public safety focus)	Informal review
General counting surveys [12]	General object counting/CAC	CNN, Transformer, foundation models	Low (general scenarios)	Informal review
Industrial vision surveys [13,14]	Defect/object detection	CNN, Transformer, deep learning	High (industrial-specific)	Informal review
Foundation model surveys	General vision foundation models	CLIP, SAM, multimodal models	Low (general domain)	Informal review
This paper	Industrial object counting	Traditional CV → CNN → Transformer → Mamba → Foundation models	High (industrial-specific)	PRISMA 2020 systematic

**Table 2:** Generalization Performance Comparison Between CLIP-based Methods and Traditional CNN Models for Counting New-Category Industrial Workpieces.

Method	Generalization Accuracy (%)	Performance Improvement (%)
CLIP-based Method: CLIP-Count [50,52]	86.3	40.2
CLIP-based Method: VLCounter [51]	87.2	41.5
Traditional CNN: CSRNet [1,47]	61.5	–
Traditional CNN: MCNN [6,47]	59.8	–

*Note*: Test datasets are NWPU-MOC and WPCD-DATASET (industrial workpiece datasets).

**Table 3:** Computational Efficiency Comparison of MCNN and Mainstream Density Map Baselines.

Model	Params	FLOPs	FPS	Architecture Type
	(M) ↓	(G) ↓	(@512×512) ↑	
MCNN	17.2	128.4	67	Multi-column CNN
CSRNet (Classic Density Baseline)	16.2	68.7	99	Single-column CNN
SFCN (Industrial Common Baseline)	8.7	35.2	165	Single-column CNN
Single-column Baseline (Same Depth as MCNN)	5.2	42.6	142	Single-column CNN

*Note*: ↓ indicates smaller values correspond to higher computational efficiency; ↑ indicates larger values correspond to faster inference speed.

**Table 4:** Main Public Datasets for Industrial Object Counting and Category Division.

Dataset Name	Dataset Type	Images Number	Categories	Annotation Type	Release Year	Publication Venue
OIRDS	General Traffic	∼900	Overhead vehicle	Bounding box	2009	IEEE
ShanghaiTech	General Crowd	1198	Crowd	Point/Density map	2016	IEEE
CARPK	General Traffic	1448	Parking lot car	Bounding box	2017	ICCV
UCF-QNRF	General Crowd	1535	Crowd	Point/Density map	2018	ECCV
VisDrone-DET2018	General Remote Sensing	8599	Human, vehicle, etc.	Bounding box	2018	ECCV
RSOC	General Remote Sensing	3057	Buildings, ships, vehicles	Point/Bounding box	2020	IEEE
NWPU-Crowd	General Crowd	5109	Crowd	Point/Density map	2021	IEEE
FSC147	General Few-Shot	6146	Daily objects, 147 categories	Bounding box/Point	2021	CVPR
FSCD-147	General Few-Shot	6146	Daily objects, 147 categories	Density map	2022	ECCV
FSCD-LVIS	General Few-Shot	6196	Common objects, 377 categories	Density map	2022	ECCV
CountBench	General Benchmark	540	–	Point	2023	ICCV
WPCD-DATASET	Industrial Exclusive	121,475	Various industrial workpieces	Bounding box/Point	2024	IEEE
BIKE-1000	General Traffic	1000	Shared bikes	Bounding box	2024	–
NWPU-MOC	Industrial Remote Sensing	3416	Aircraft, ships, vehicles, farmland facilities, etc.	Point/Density map	2024	IEEE
MCAC	General Multi-Class	16,224	Common daily objects	Point/Density map	2024	ECCV
OmniCount-191	General Multi-Class	30,230	Kitchenware, office supplies, transportation tools, etc.	Density map/Semantic mask	2025	AAAI

Note: “Industrial Exclusive” and “Industrial Remote Sensing” datasets are constructed for industrial production and industrial remote sensing scenarios, which can be directly used for training and evaluation of industrial counting algorithms; “General” datasets are oriented to general scenarios such as crowd, traffic and daily objects. When applied to industrial counting tasks, cross-domain transfer processing is required. “–” indicates that the relevant information is not publicly available.

## Data Availability

No new data were created or analyzed in this study. Data sharing is not applicable to this article.

## Abbreviations

The following abbreviations are used in this manuscript:

CNN	Convolutional Neural Network
CAC	Class-Agnostic Counting
MAE	Mean Absolute Error
RMSE	Root Mean Square Error
NMS	Non-Maximum Suppression
FCN	Fully Convolutional Network
MCNN	Multi-Column Convolutional Neural Network
DETR	DEtection TRansformer
SSM	State Space Model
CLIP	Contrastive Language-Image Pre-training
SAM	Segment Anything Model
FSC	Few-Shot Counting
ZSC	Zero-Shot Counting
EFC	Exemplar-Free Counting
